# Single-cell analysis of graft-infiltrating host cells identifies caspase-1 as a potential therapeutic target for heart transplant rejection

**DOI:** 10.3389/fimmu.2023.1251028

**Published:** 2023-09-13

**Authors:** Zhichao Wu, Jialiang Liang, Shuoji Zhu, Nanbo Liu, Mingyi Zhao, Fei Xiao, Guanhua Li, Changjiang Yu, Chengyu Jin, Jinshan Ma, Tucheng Sun, Ping Zhu

**Affiliations:** ^1^ Guangdong Cardiovascular Institute, Guangdong Provincial People’s Hospital (Guangdong Academy of Medical Sciences), Southern Medical University, Guangzhou, Guangdong, China; ^2^ Guangdong Provincial Key Laboratory of Pathogenesis, Targeted Prevention and Treatment of Heart Disease, Guangzhou Key Laboratory of Cardiac Pathogenesis and Prevention, Guangzhou, Guangdong, China; ^3^ Department of Thoracic Surgery, People’s Hospital of Xinjiang Uygur Autonomous Region, Urumqi, Xinjiang, China

**Keywords:** single cell RNA and transcriptome sequencing, mouse heart transplantation model, immune infifiltration landscape, interferon signaling, inflammasome, allograft rejection

## Abstract

**Aims:**

Understanding the cellular mechanisms underlying early allograft rejection is crucial for the development of effective immunosuppressant strategies. This study aims to investigate the cellular composition of graft-infiltrating cells during the early rejection stage at a single-cell level and identify potential therapeutic targets.

**Methods:**

A heterotopic heart transplant model was established using enhanced green fluorescent protein (eGFP)-expressing mice as recipients of allogeneic or syngeneic grafts. At 3 days post-transplant, eGFP-positive cells infiltrating the grafts were sorted and subjected to single-cell RNA-seq analysis. Potential molecular targets were evaluated by assessing graft survival and functions following administration of various pharmacological inhibitors.

**Results:**

A total of 27,053 cells recovered from syngrafts and allografts were classified into 20 clusters based on expression profiles and annotated with a reference dataset. Innate immune cells, including monocytes, macrophages, neutrophils, and dendritic cells, constituted the major infiltrating cell types (>90%) in the grafts. Lymphocytes, fibroblasts, and endothelial cells represented a smaller population. Allografts exhibited significantly increased proportions of monocyte-derived cells involved in antigen processing and presentation, as well as activated lymphocytes, as compared to syngrafts. Differential expression analysis revealed upregulation of interferon activation-related genes in the innate immune cells infiltrating allografts. Pro-inflammatory polarization gene signatures were also enriched in these infiltrating cells of allografts. Gene profiling and intercellular communication analysis identified natural killer cells as the primary source of interferon-γ signaling, activating inflammatory monocytes that displayed strong signals of major histocompatibility complexes and co-stimulatory molecules. The inflammatory response was also associated with promoted T cell proliferation and activation in allografts during the early transplant stages. Notably, caspase-1 exhibited specific upregulation in inflammatory monocytes in response to interferon signaling. The regulon analysis also revealed a significant enrichment of interferon-related motifs within the transcriptional regulatory network of downstream inflammatory genes including caspase-1. Remarkably, pharmacological inhibition of caspase-1 was shown to reduce immune infiltration, prevent acute graft rejection, and improve cardiac contractile function.

**Conclusion:**

The single-cell transcriptional profile highlighted the crucial role of caspase-1 in interferon-mediated inflammatory monocytes infiltrating heart transplants, suggesting its potential as a therapeutic target for attenuating rejection.

## 1 Introduction

Heart transplantation is the ultimate treatment for end-stage heart failure or injuries that are unresponsive to medical or surgical interventions (1). A major advance in the development of immunosuppressants contributes to reductions in acute organ rejection and improvements in survival outcomes by targeting key alloreactive T-cell (TC) mechanisms (2). However, the transplanted organs would fail within years after surgery due to cardiac allograft vasculopathy (3). Emerging evidence supports that innate immune responses play a crucial role in the occurrence of adaptive immunity and chronic rejection, therefore representing a valuable target to further improve long-term allograft survival (4, 5). Thus, the development of new therapeutics for mitigating rejection requires a fundamental understanding of the immunobiological mechanisms underlying innate immune responses.

Innate immune responses play a crucial role in the occurrence of adaptive immunity and chronic rejection, therefore representing a valuable target to improve allograft survival (4, 5). Immune cell infiltration in the donor’s graft is the major pathologic hallmark of transplant rejections (6). Recipient-derived innate immune cells are prominent cell types for TC activation and organ rejection (7). Interactions between the donor’s cells and the recipient’s immune system contribute to the initiation of allograft rejection through direct, indirect, or semidirect pathways of allorecognition (8). Therefore, manipulation of the recipient’s immune responses at an early stage is a promising strategy to avoid chronic rejection. Due to heterogeneous immune populations maintaining a delicate balance of rejection and tolerance (5, 9), high-resolution mapping of the cell landscape is essential to identify novel therapeutic targets for preventing chronic rejection.

Single-cell RNA sequencing (scRNAseq) has emerged as a powerful tool to characterize cellular heterogeneity in heart development and various heart disease models (10, 11). Currently, scRNAseq has been utilized in studies of murine models of heart transplantation (12–16), leading to the identification of cell subpopulations or signaling pathways associated with rejection. However, the cell pooling approaches used in these studies cannot directly distinguish between infiltrating host cells and donor resident cells. Understanding how recipient/host cells recognize nonself molecules and trigger infiltration, especially during the early stages of heart transplantation, is crucial not only for gaining insights into the underlying mechanisms but also for the development of novel therapeutics. Moreover, there is a lack of comprehensive single-cell analysis examining the full range of infiltrates including both immune and non-immune cells. Thus, it is worthwhile to re-examine cell populations involved in rejection using a cell tracking model which allows for the identification of new cell states and intercellular networks through scRNAseq.

In this study, we utilized transgenic mice with ubiquitous enhanced green fluorescent protein (eGFP) expression as recipients to identify infiltrates in heart grafts and gain insights into the early immune events during acute rejection by scRNAseq. To the best of our knowledge, this is the first comprehensive study analyzing graft-infiltrating host cells tracked with the transgenic reporter through scRNAseq. The communication between the innate and adaptive immune systems is facilitated by the activation of inflammation and interferon (IFN)-induced signaling pathways in monocytes. These pathways play an essential role in the development of rejection and graft dysfunction in heart allograft recipients. Our study provides a proof-of-concept for the therapeutic potential of targeting inflammation-related components such as caspase-1 to reduce rejection and improve graft function.

## 2 Methods

More detailed materials and methods are available in the supplemental information.

### 2.1 Animals

All animal protocols and experiments were reviewed and approved by the Medical Ethics Committee of Guangdong Provincial People’s Hospital, Guangdong Academy of Medical Sciences. Male eGFP, C57BL/6J, or BALB/c mice at 10–12 weeks of age with a body weight of 25-28 g were used for this study. Wild-type mice and eGFP mice were purchased from the Jackson Lab (Stock No: 006567). All animals were maintained in a pathogen-free environment. Commercial chow and tap water were made available ad libitum. All animals received humane care in compliance with the ‘Principles of Laboratory Animal Care’ formulated by the National Society for Medical Research and the ‘Guide for the Care and Use of Laboratory Animals’ prepared by the Institute of Laboratory Animal Resources and published by the National Institutes of Health (NIH Publication No. 86-23, revised 1996).

### 2.2 Heart transplant models

The surgery of murine heterotopic heart transplant was performed according to the previous study (17). All animals were anesthetized with an intraperitoneal injection of 1% ketamine and 0.2% xylene and then placed supine on the operative field. The donor’s heart was isolated as follows. After the midline abdominal incision, 0.3 mL of 100 Unit/mL ice-cold heparin sodium was slowly injected into the donor mice at the inferior vena cava (IVC) using an insulin syringe. Under systemic heparinization, the thoracotomy was performed and the chest was opened. The heart was wrapped with ice-cold surgical gauze. The superior vena cava (SVC), inferior vena cava (IVC), and azygos veins, azygos vein, aorta, pulmonary artery, and pulmonary veins were then ligated and cut off to free the heart that was perfused with cold HTK solution to remove the blood and kept in cold HTK solution until transplant. The abdomen of the recipient mouse was opened with a midline incision, and a length of the abdominal aorta and IVC between the renal vessels and iliac bifurcation was freed. 6-0 silk was placed under both sides of the abdominal aorta and the IVC. The spinal veins were then ligated with 6-0 silk, and a slipknot was made to block the flow of blood to the abdominal aorta and the IVC. Finally, the donor’s ascending aorta and pulmonary artery were anastomosed to the recipient’s abdominal aorta and inferior vena cava, respectively, using 11-0 sutures. Finally, 4-0 sutures were used for closing the recipient’s abdomen. Cardiac allograft survival was determined by daily palpation and binocular inspection.

### 2.3 Single-cell dissociation

At 3 days after heart transplantation, the extravascular immune cells were isolated using enzymatic and mechanical digestion as previously described (18). To avoid blood coagulation, systemic heparinization was induced with an intraperitoneal injection of 1 mL of 100 Unit/mL heparin sodium for 10 min. Subsequently, the recipients were euthanized using a CO_2_ chamber and the abdomen was reopened to expose the implanted heart. The right and left atria were removed and avoided injuring arteries or veins. The heart base was gently grasped with forceps and the 21 G needle was inserted into the left ventricle near the apex. The heart was perfused with 15-20 mL of cold PBS using a syringe. And the right ventricle was also perfused using the same procedure. Then, the heart graft was surgically excised from the abdominal aorta and inferior vena cava, and its ascending aorta and pulmonary artery with anastomosed sites were cut off. The explanted heart was rinsed with-cold PBS and then collected in the ice-cold DMEM media (Gibco) containing 10% fetal bovine serum (Corning). The heart was placed in a 5 cm plastic dish with 50 µL of cold media and minced using dissecting scissors until there were no visible pieces. The tissue slurry was transferred to a collection tube and digested using 450 U/mL of collagenase I (Thermo Fisher Scientific), 60 U/mL of hyaluronidase type I-S (Thermo Fisher Scientific), and 60 U/mL of DNase-I (Thermo Fisher Scientific) and incubate at 37°C on a rocking shaker at 50 rpm for 60 min. After digestion, the homogenized sample was filtered with a 40-mm cell strainer (Corning) and transferred into a 50 mL conical tube with 1 mL of cold HBB (Gibco). Finally, the single−cell suspension was treated with 1 mL of Ammonium-Chloride-Potassium (ACK) buffer lysis (Thermo Fisher Scientific) to lyse red blood cells, centrifuged samples at 400 g for 5 min at 4°C, and resuspended with cold HBB for cell staining and sorting.

### 2.4 Fluorescence−activated cell sorting

The single-cell suspension was incubated with 1 µL SYTOX™ Blue dead cell stain (Thermo Fisher Scientific) to exclude the dead cells and then analyzed and sorted by FACS Aria II cell sorter (BD Biosciences). The eGFP reporter signal was measured with the fluorescein isothiocyanate (FITC) channel, while SYTOX™ Blue was detected with the DAPI channel. The FACS imaging was analyzed using the FlowJo v10 software. Finally, eGFP^+^ and SYTOX™ Blue^-^ cells with high viability (>92%) were sorted for scRNAseq.

### 2.5 Single-cell RNA sequencing

Single-cell suspensions (~1000 cells/uL) were loaded on the Chromium Single cell Controller (10×Genomics) to generate a single cell and gel bead emulsion (GEM) according to the manufacturer’s instruction. scRNA-seq libraries of the single cells from syngenic or allogenic heart graft pools were generated by the Single Cell 5’v1 with V(D)J Enrichment Kit (mouse T cell receptor (TCR) or B cell receptor (BCR)), while the additional replicates were profiled using the Single Cell 3’v3 assays. After reverse transcription, GEMs were broken and single-strand cDNA was purified and amplified with the thermal cycler. Subsequently, the amplified barcoded cDNA was cleaned up, fragmented, poly A-tailed, ligated with adaptors, and index PCR amplified. The final libraries were quantified using the Qubit High Sensitivity DNA assay (Thermo Fisher Scientific) and the size distributions of the libraries were determined using a High Sensitivity DNA chip on the Agilent Bioanalyzer 2200 for quality control. All libraries were sequenced by the NovaSeq 6000 sequencer (Illumina) on a 150 bp paired-end run. Detailed methods for downstream sequencing analysis can be found in the supplemental information section.

### 2.6 Data availability

All single-cell sequencing raw data of this study were deposited in the Genome Sequence Archive of the China National Center for Bioinformation (accession number CRA007855) and are publicly accessible at https://bigd.big.ac.cn/gsa/browse/CRA007855. The codes and other resources used in this study are available from the corresponding author upon reasonable request.

### 2.7 Statistical analysis

For scRNAseq data, the involved statistical analysis was performed in the bioinformatics tools as described above. Statistical significance was accepted for *p*-value < 0.05. For non-scRNAseq data, statistical analysis was performed using GraphPad Prism 10 Software. The group comparison in survival analysis was performed using the Gehan-Breslow-Wilcoxon test. Differences between two mean values were evaluated by an unpaired Student’s t-test, while the data of multiple groups were tested for statistical significance using one-way ANOVA followed by Bonferroni’s *post hoc* analysis. All graphic data were presented as mean ± standard error. A *p*-value of <0.05 was considered statistically significant.

## 3 Results

### 3.1 An unbiased single-cell landscape of recipient-derived infiltrates

**Figure 1:**
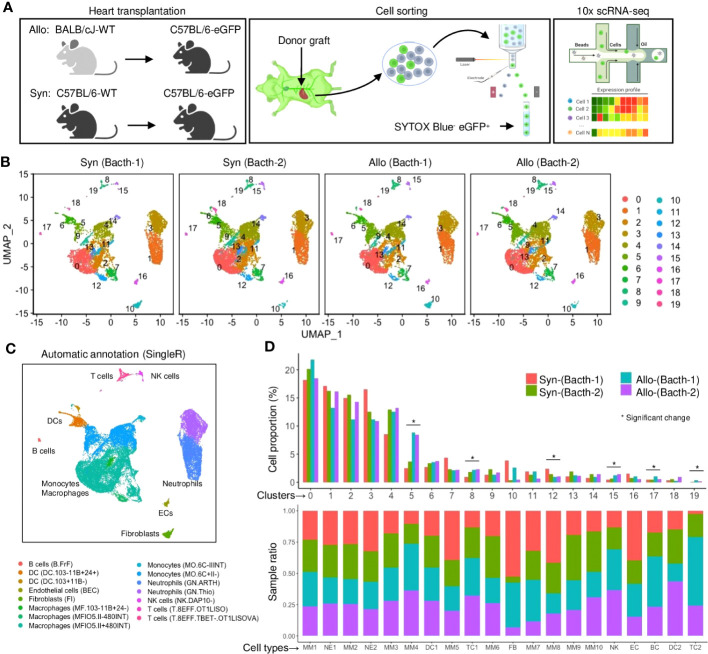
A single-cell landscape of infiltrating host cells in heart grafts. **(A)** Schematic of experiment design including the mouse groups of heart transplantation, fluorescence-activated cell sorter (FACS) strategy, and single-cell RNA sequencing (scRNA-seq) platform. The β-actin-driven eGFP is constitutively expressed in all cells including immune or non-immune cells (except erythrocytes) derived from the recipient. **(B)** Uniform manifold approximation and projection (UMAP) plots of 27,053 cells collected from four samples with each cell color-coded for its cell cluster number. **(C)** UMAP plot of all cells with phenotype annotations. Color is coded according to the SingleR annotation (using the ImmuGen dataset). **(D)** Bar plots of the proportions of cells in each of the 20 identified cell populations (upper) and the fraction of each cluster originating from different samples (lower). Colored by the sample source. * FDR < 0.05 and fold change > 1.5 (scProportionTest).

Heterotopic cardiac transplant models were established using eGFP^+^ C57BL/6 mice as recipients of allogeneic BALB/c (Allo) or syngeneic C57BL/6 (Syn) grafts (**Figure 1A**). Flow cytometry showed that the eGFP^+^ cell percentage of allografts was low on day-3 but increased markedly after 5 days as compared to syngrafts (**Figures S1A, B**). Subsequently, live eGFP^+^ cells were sorted for scRNAseq on day-3 post-transplant representing the early stages of the alloimmune response (**Figure S1C**). To enhance robustness and reduce variability, duplicates of Syn or Allo pooling heart tissues were analyzed by the single-cell 5’v1 (Batch-1) and 3’v3 (Batch-2) assays (**Figure S1D**). Sequencing data were quality controlled using the Cell Ranger (**Table S1**) and 30,092 cells were recovered for further analysis. To minimize batch effects from different chemistry libraries (**Figure S1E**; **Table S2**), we reconstructed datasets using the Seurat package (19). After the Seurat object generation and dimensionality reduction for each library, DoubletFinder (20) was used to filter out the potential doublets, and the final 27,053 cells (singlets) from both syngrafts and allografts were used for the downstream analysis (**Figure S1F**).

Unsupervised Seurat clustering was performed on all samples’ normalized and integrated datasets to maximize detection resolution. The clustering tree analysis indicated a resolution of 0.3 with few numbers of low in-proportion edges, which was chosen to classify 20 cell subsets across the four samples based on gene similarity (**Figure S2A**). 2D projected maps showed that cell distribution was comparable among all samples, excluding batch effects (**Figure 1B**). The positive marker genes were profiled through the Seurat differential expression analysis between a cluster and all other cells (**File S1**). Most clustered cells showed distinguishable patterns of the highly expressed marker genes irrespective of treatment conditions (**Figure S2B**), while the hierarchical clustering of Pearson correlation analysis showed several clusters with similar transcriptomic profiles (**Figure S2C**).

### 3.2 Annotation of graft-infiltrating cells

Next, the cell clusters were biologically interpreted using the reference-based SingleR annotation (21), as labeled on a heatmap of confidence scores (**Figure S2D**). Therefore, the Seurat clusters were re-categorized as 9 main cell types or 15 subtypes by the SingleR analysis (**Figures 1C**, **S2E**). Notably, over 90% of infiltrates were comprised of myeloid immune cells (including monocytes and macrophages (MMs), neutrophils (NEs), and dendritic cells (DCs)) in syngrafts or allografts (**Figure 1D**; **Table S3**), suggesting the predominant role of innate immunity in this stage. The proportions of several lymphocyte clusters (such as TCs and B cells (BCs)) were increased in allografts as analyzed by a permutation test (**Figure S2F**), indicating the onset of adaptive immune responses. Several eGFP^+^ populations contained cells with low Cd45 (*Ptprc*) expression (**Figures S1A**, **S2G-H**), suggesting that the eGFP^+^ cell isolation may capture a more comprehensive landscape of recipient-derived infiltrates than the sorting approach using Cd45 antibodies. Further, the cell annotations were confirmed by the gene ontology (GO) and Kyoto encyclopedia of genes and genomes (KEGG) analysis of differentially expressed genes (**Figures S3A, B**). Several cell types shared cell migration, chemotaxis, proliferation, or antigen processing pathways, while unique pathways were identified to characterize the cell functions.

### 3.3 Monocytes and macrophages are predominant infiltrates

**Figure 2:**
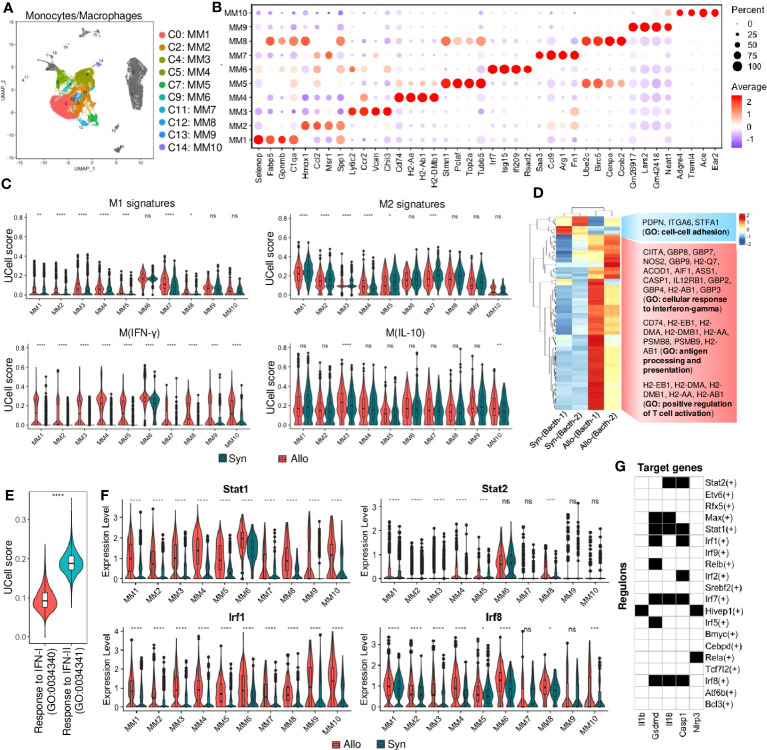
Monocyte and macrophage cell responses. **(A)** UMAP plot showing distributions of monocytes and macrophages (MMs). Color is coded according to their associated Seurant cluster. **(B)** A dot plot showing the representative gene expressions in each MM subset. The scale of dot size representing cell percentage and dot color representing the average expression is shown on the right. The prominently expressed marker genes are highlighted by the red color. **(C)** Violin plots showing the UCell signature scores of representative macrophage activation (M1 to M2) states in MM subsets from syngrafts or allografts. **(D)** Heatmap showing the differential gene expressions of pseudobulk samples by the DESeq2 analysis. And gene ontology (GO) terms related to the up-regulated or down-regulated genes are shown on the right. **(E)** Violin plots showing the UCell gene signature scores of the response to type-I interferon (IFN-I) and type-II interferon (IFN-II) GO terms in bulked MM subsets from heart grafts. **(F)** Violin plot showing normalized expression levels of the interferon-related transcription factors in MM subsets from syngrafts or allografts. ^ns^ not significant, * *p*<0.05, ** *p*<0.01, *** *p*<0.001, **** *p*<0.0001 (Student’s t-test). **(G)** A heatmap showing the top transcription factors/regulons (rows, from SCENIC-RSS analysis) for inflammation-related genes (columns) in monocytes and macrophages. Back color indicates TF motif-targeting and white color indicates non-targeting.

The identified MMs shared common markers such as Cd68, Cd115, and F4/80 (**Figure S4A**) but were composed of various populations or stages. MMs were the largest cell infiltrate population in syngrafts or allografts (>50%, **Figure 1D**; **Table S3**). Furthermore, individual MM subtypes 1-10 were reanalyzed according to the highly expressed genes that are associated with inflammation, antigen processing, or cell cycling (**Figures 2A, B**; **Table S4**). Further, the UCell (22) analysis with a list of marker gene sets (from a nomenclature guideline (23)) was performed to define the macrophage activation states (**Figure 2C**). MM6 exhibited the M1 or M(IFN-γ, or IFN-II) pro-inflammatory phenotypes, while others were close to the M2 or M(IL-10) anti-inflammatory phenotypes. Activation of the M(IFN-γ) genes indicated the pro-inflammatory macrophage transition in allografts.

The gene expression profiles for the main cell types were further summarized at the population level by aggregating them into a pseudobulk using DESeq2 (24) (**File S2**). The MMs were extracted for differential expression analysis between syngrafts and allografts. The most variable genes were analyzed by hierarchical clustering to determine the biological reproducibility of sample replicates (**Figure 2D**). The genes upregulated in allografts were related to the cellular response to IFN-γ, antigen processing and presentation, and positive regulation of TC activation, while the down-regulated genes were related to the GO term of cell-cell adhesion (**Figure S4B**). Interestingly, the GO terms related to IFN-β and IFN-γ signaling were simultaneously enriched in the allograft group (**Figure S4B**). UCell analysis was further conducted to assess the distinct aspects of the immune response to IFN-I and IFN-II. Notably, the gene signature score of IFN-II was significantly higher than that of IFN-I in the MM populations (**Figure 2E**). GSEA also indicated that significant genes were involved in the KEGG pathways such as allograft rejection, antigen processing and presentation, and graft-versus-host disease (**Figure S4C**). Therefore, IFN signaling in infiltrating MMs may contribute to alloimmunity at 3 days post-heart transplant.

Single-cell regulatory network inference and clustering (SCENIC) (25) with regulon specificity score (RSS) (26) was performed to assess transcription factors (TFs) responsible for the differential gene expression between syngrafts and allografts (**Figures S4D, E**). MMs were further re-clustered based on the binarized regulon activity matrix (**Figure S4D**), as shown in a heatmap highlighting the top 20 specific TFs of syngrafts or allografts. Most of the up-regulated genes identified by the pseudobulk analysis were co-expressed with the top TFs in allografts rather than syngrafts as shown in the regulon incidence matrix of TF-target networks (**Figure S4F**). For instance, the IFN-II-related TFs (e.g., *Irf1* and *Irf8*) *(* 27) were up-regulated in allografts, accompanied by high expressions of the downstream genes (e.g., *Gbp2*, *Gbp4*, and *Igtp*) *(* 28) (**Figures 2F**, **S4G**). It was also observed that the expression of IFN-I-related TFs and genes (e.g., *Stat2*, *Irf7*, and *Isg15*) *(* 29, 30) remained low (the normalized and log-transformed value<1.0) in the majority of MMs, despite notable differences between syngrafts and allografts (**Figures 2F**, **S4G**). Remarkably, certain downstream TFs shared by both IFN-I and IFN-II pathways (e.g., *Stat1* and *Samhd1*) *(* 30, 31) exhibited high expression levels in allograft-infiltrating MMs and demonstrated significant distinctions in comparison to syngrafts (**Figures 2F**, **S4G**). Furthermore, the SCENIC-based regulon analysis revealed a significant enrichment of IFN-related TF motifs (such as Stat1, Stat2, Irf1, Irf7, and Irf8) within the transcriptional regulatory network of downstream inflammatory genes (**Figure 2G**). Hence, the intricate interplay between IFN pathways and TF activation in MMs plays a pivotal role in the development and progression of allograft inflammation.

### 3.4 Monocyte-derived antigen-presenting cells are enriched in allografts

**Figure 3:**
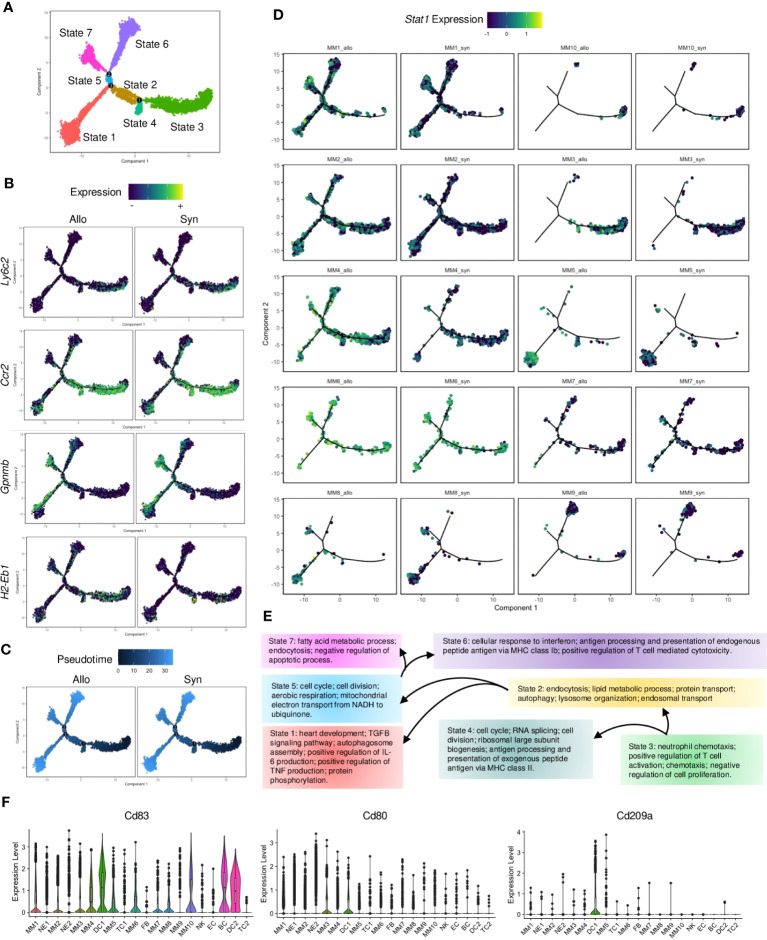
Pseudotime analysis of monocytes and macrophages. **(A)** Monocle2 trajectory plot showing potential paths of monocyte and macrophage differentiation, colored according to the cell state. **(B)** Trajectory plots showing the expression level of the *Ly6c2*, *Ccr2*, *Gpnmb*, and *H2-Eb1* genes in the MM subsets. The average expression scale is shown on the top. **(C)** Trajectory plots showing pseudotime distribution of MM subsets split by the graft types. The time scale is shown on the top. Pseudotime-0 indicates starting cells. **(D)** Trajectory plots showing the *Stat1* gene expression split by each MM subset from different graft types. The average expression scale is shown on the top. **(E)** Flowchart of cells making fate choices of cell states along the 3 trajectory branch points referred to **(A)**. And the representative GO terms related to cell state-specific genes are listed. **(F)** Violin plots showing normalized expression levels of the APC markers (*Cd83*) or DC markers (*Cd80* and *Cd209a*) in all cell subsets.

Potential cellular dynamics of MM transition were delineated using the Monocle (32) trajectory analysis. The MMs were re-categorized as 7 states (**Figure 3A**). Circulating monocyte markers (e.g., *Ly6c2* and *Ccr2*) were expressed in cell state-3, whereas differentiated macrophage markers (e.g., *Gpnmb (* 33)) were upregulated in other cell states along this trajectory (**Figure 3B**). APC markers (e.g., *H2-Eb1*) were also expressed differentially in the cell trajectories of syngrafts and allografts. MM3 (highly expressing *Ly6c2*) was assumed as the root of cell trajectory, and the transition state-3 was shown as the initial cell state of the pseudotime tree in both syngrafts and allografts (**Figure 3C**). The trajectory map was split to dissect cell heterogenesis in grafts (**Figure 3D**). Most MM3 cells were enriched in state-3, while other cell types were scattered in more than 2 cell states suggesting undergoing transition states. Remarkably, *Stat1* was up-regulated in allografts across different cell states as compared with syngrafts. Gene variations of state transition across tree branches were summarized according to the GO terms (**Figure 3E**). The initial state-3 was associated with chemotaxis, while the intermediate states 2, 4, and 5 modulated the metabolic status or cell cycle. The terminal states 1, 6, and 7 were associated with self-repair, antigen processing, and fatty acid process, respectively.

Notably, MM4 was the only MM population that significantly increased (**Figure S2F**) and enriched at the transition states 6 and 7 in allografts as compared to syngrafts (**Figure 3D**). It was derived from circulating monocytes as shown by *eGFP*, *Ptprc*, and *Itgam* (**Figure S5A**). MM4 was expressed with APC or macrophage genes but not canonical DC markers (e.g., *Cd103*, *Cd80*, and *Cd209a*) (**Figures 3F**, **S5A**), therefore classified as monocyte-derived cells based on the nomenclature (34). IFN-related genes (e.g., *Stat1*, *Ifi47*, and *Gbp2*) were highly expressed in MM4 in allografts (**Figure S5B**). Re-analyzing with Monocle, MM4 was identified with 2 separate trajectory branches and 5 cell states based on the gene variation between syngrafts and allografts (**Figure S5C**). A cell state of MM4 that exclusively distributed in allografts was defined as the ‘Allo-state’, while the cell state in the opposite direction was termed the ‘Syn-state’ (**Figure S5C**). Further, pseudotime-dependent genes were clustered in a heatmap comparing the two states (**Figure S5D**). The genes associated with IFN-β response, TC cytotoxicity, or antigen processing exhibited an increasing trend in the Allo-state of the MM4 subset (**Figures S5E, F**).

### 3.5 Involvement of dendritic cells or neutrophils in the early transplant stage

**Figure 4:**
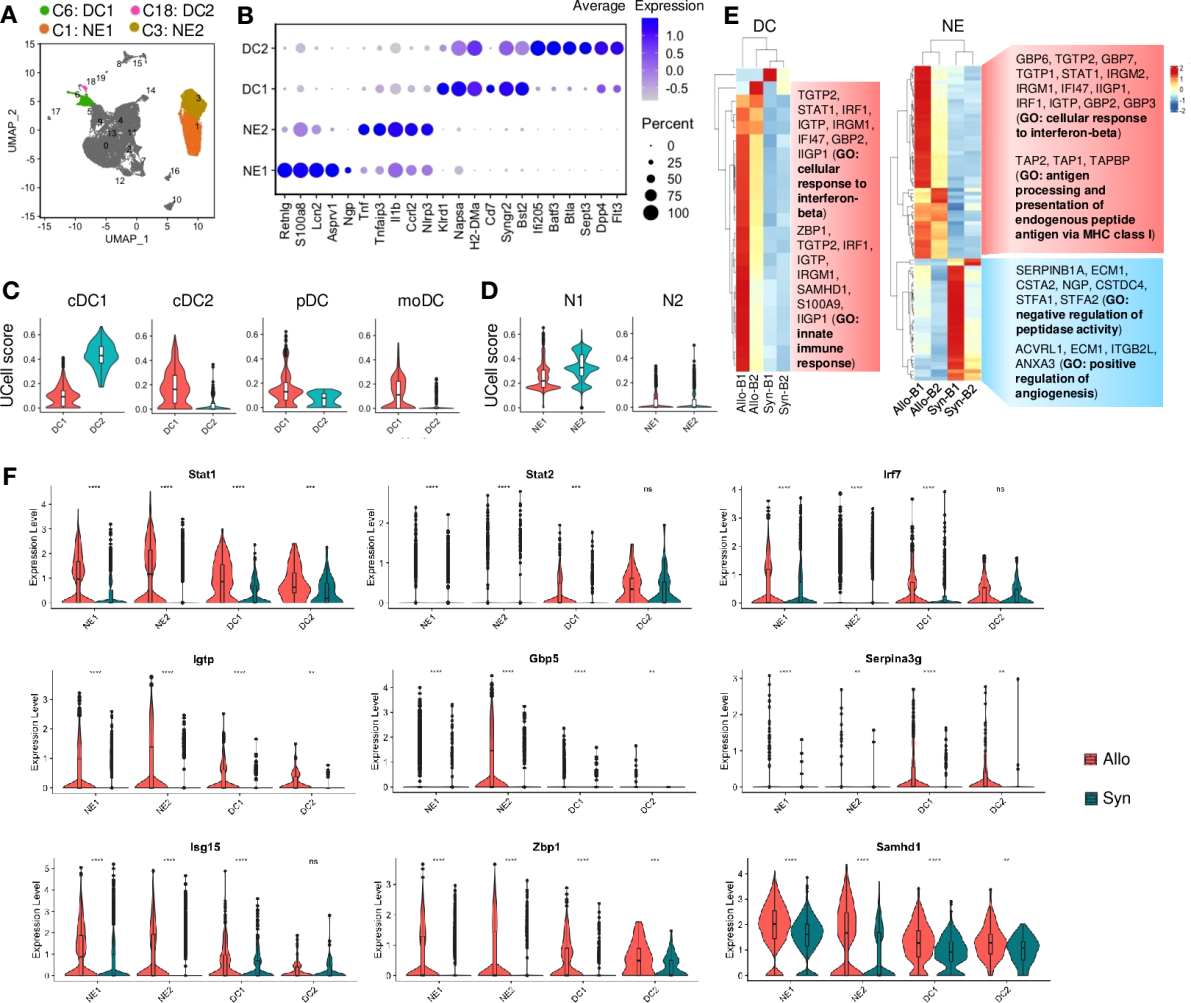
Neutrophil and dendritic cell activities. **(A)** UMAP plot showing distributions of neutrophils and dendritic cells. Color is coded according to their associated Seurant cluster. **(B)** A dot plot showing the representative gene expressions in neutrophils and dendritic cells. The scale of dot size representing cell percentage and dot color representing the average expression is shown on the right. **(C)** Violin plots showing the UCell gene signature scores of defined dendritic cell types. cDC1: type 1 conventional dendritic cells; cDC2: type 2 conventional dendritic cells; pDC: plasmacytoid dendritic cells; moDC: monocyte-derived DCs. **(D)** Violin plots showing the UCell gene signature scores of neutrophil activation states. N1: pro-inflammatory neutrophil; N2: anti-inflammatory neutrophil. **(E)** Heatmaps showing the differential gene expressions of pseudobulk samples by the DESeq2 analysis in dendritic cells (DC) and neutrophils (NE), respectively. And gene ontology (GO) terms related to the up-regulated or down-regulated genes are shown on the right. B1: Batch-1; B2: Batch-2. **(F)** Violin plots showing normalized expression levels of the IFN-I-related transcription factors or target genes in neutrophils or dendritic cells from syngrafts or allografts. ^ns^ not significant, ** *p*<0.01, *** *p*<0.001, **** *p*<0.0001 (Student’s t-test).

NE or DC subtypes were also identified by the differential gene expression (**Figures 4A, B**; **Table S4**). DC1 and DC2 shared with major histocompatibility complex (MHC)-II molecules and *Cd11c* but exhibited distinct transcriptional signatures. For instance, *Klrd1* and *Cd7* (involved in natural killer (NK) cell activation) (35) were upregulated in DC1 (**Figures 4B**, **S6A**), while markers (*Batf3*, *Cd103*, and *Xcr1*) of type 1 conventional dendritic cells (cDCs) were highly expressed in DC2. The UCell analysis of the well-studied 4 types of DCs also showed that DC2 was classified as cDC1, but DC1 resembled cDC2 due to the higher gene signature scores (**Figure 4C**). And the UCell scores of pDC and moDC were low in DC1 or DC2. NE1 and NE2 were identified with neutrophil markers (e.g., *S100a9*, *Csf3r*, *Il1r2*, and *Mmp9*) but showed different signatures (**Figures 4B**, **S6B**). For instance, a large number of NE1 cells were expressed with mature neutrophil markers (e.g., *Retnlg*, *Lcn2*, and *Asprv1*, associated with chronic inflammation) (36, 37), while NE2 cells were highly expressed with pro-inflammatory molecules (e.g., *Tnf*, *Il1b*, and *Nlrp3*, which trigger acute inflammation) (38) (**Figures 4B**, **S6B**). Like macrophages, NEs were shown to polarize into N1 (similar to M1) or N2 (similar to M2) phenotypes under pathological conditions (39, 40). The UCell score analysis showed that both NE1 and NE2 mainly resembled the N1 phenotype (**Figure 4D**).

Although there was no difference in the cell proportion of DCs or NEs (**Figure S2F**), we conducted the pseudobulk analysis to evaluate potential variations in gene expression profiles. The variable genes of cellular response to IFN-β and innate immune response were upregulated in allograft DCs and NEs as shown by hierarchical clustering (**Figure 4E**). Despite a few differentially expressed genes (fold change>2, *padj*<0.05) in DCs or NEs (**File S2**), GSEA indicated that the top KEGG pathways in DCs or NEs had no difference between allografts and syngrafts (**Figures S6C, D**). Additionally, the SCENIC with RSS indicated the top 5 TFs in DCs or NEs (**Figure S6E**). The target genes up-regulated in DCs or NEs were co-expressed with the top TFs in allografts rather than syngrafts (**Figure S6F**). The IFN-I-related TFs such as *Stat1*, *Stat2*, and *Irf7* were up-regulated in allograft DCs or NEs, accompanied by high expressions of the downstream target genes (*Gbp5*, *Igtp*, and *Serpina3g*) (**Figures 4F**, **S6F**). Additionally, other IFN-I-related genes such as *Isg15*, *Zbp1*, and *Samhd1* were up-regulated in allograft DCs or NEs (**Figure 4F**). Therefore, the gene activation of IFN-I signaling in DCs or NEs was also crucial for alloimmunity development.

### 3.6 Activation of lymphocytes during the early transplant stage

**Figure 5:**
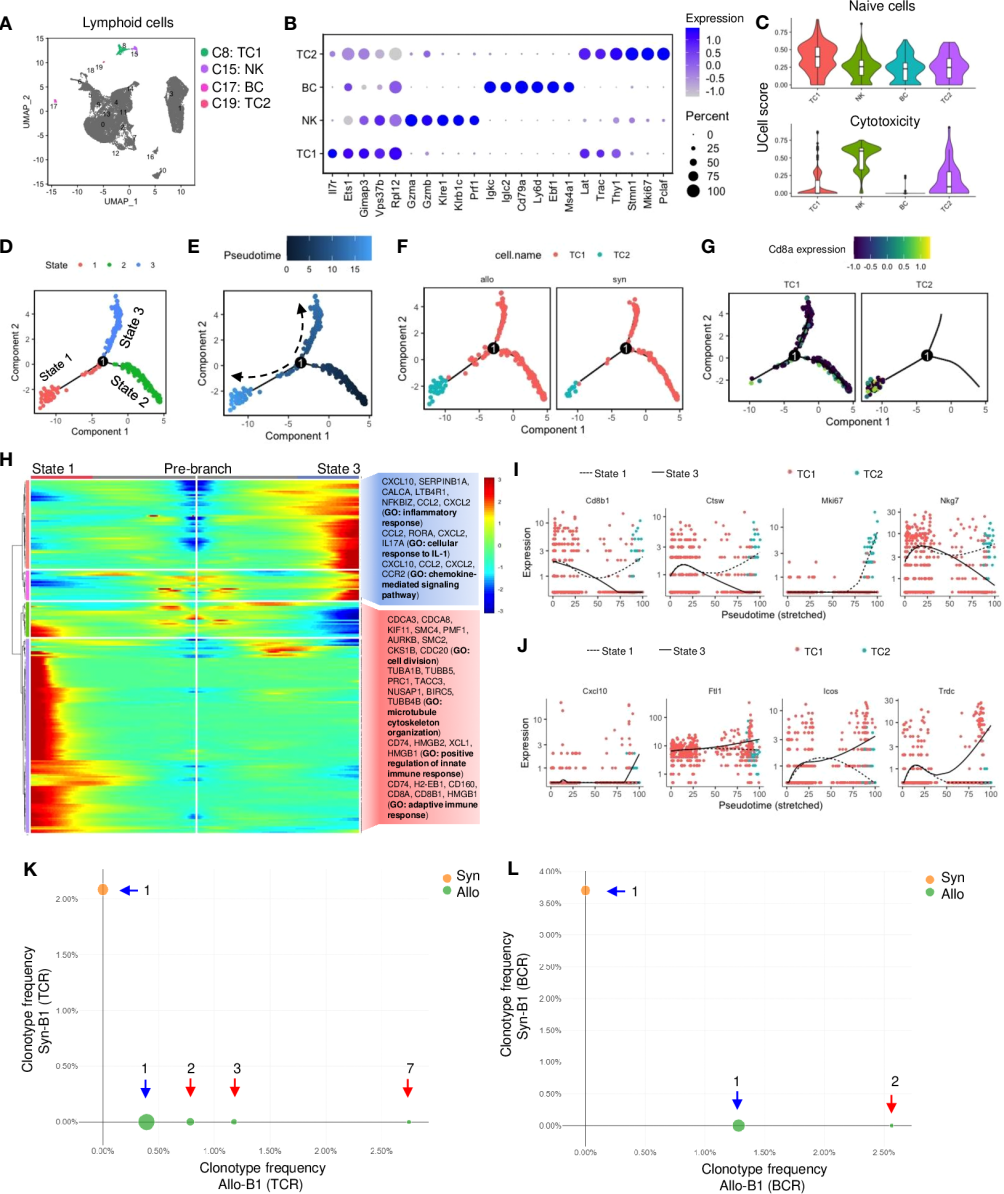
Activation of T and B lymphocytes and natural killer cells in allografts. **(A)** UMAP plot showing distributions of T cells, B cells, and natural killer cells. Color is coded according to their associated Seurant cluster. **(B)** A dot plot showing the representative gene expressions in T cells, B cells, and natural killer cells. The scale of dot size representing cell percentage and dot color representing the average expression is shown on the right. **(C)** Violin plots showing the UCell gene signature scores of naive state or cytotoxic activity. **(D)** Trajectory plot showing potential paths of T cell activation, colored according to the cell state. **(E)** Trajectory plots showing pseudotime distribution of T cells. The time scale is shown on the top. Pseudotime-0 indicates starting cells. **(F)** Trajectory plots showing the distribution of T cells split by different graft types. Color is coded according to the cell type. **(G)** Trajectory plots showing the *Cd8a* gene expression split by each T cell subset. Average expression scale is shown on the top. **(H)** Heatmap showing lineage-dependent gene expression patterns by comparing the state-1 and state-3 split from the branch point-1 **(D)**. And gene ontology (GO) terms related to the gene profiles are shown on the right. **(I, J)** Scatter plots of the expression levels of representative genes upregulated or downregulated along the pseudotime. Color is coded according to the cell type. The solid line indicates state-3. Dotted line indicates state-1. **(K, L)** Loupe VDJ Browser analysis of T cell receptor (TCR)-V(D)J sequencing of T cell clonotypes or B cell receptor (BCR)-V(D)J sequencing of B cell clonotypes from syngrafts and allografts as indicated by two colors. The number of clonal expansions is shown. The circle size at each point of frequency represents the number of clonotypes. Blue arrow indicates singleton clonotypes and red arrow indicates expanded clonotypes.

Lymphoid cells, including TC subtypes 1-2, BC, and NK cells, were also identified (**Figure 5A**; **Table S4**). Although these cells constituted a small population (~5%) of infiltrates (**Table S3**), their proportions were significantly increased in allografts (**Figure S2F**). They were specifically expressed with *Ptprcap* which was absent in myeloid cells (**Figure S7A**). *Cd3* was exclusively expressed in TCs and the activation markers (e.g., *Il17r*, *Lat*) were also highly expressed (**Figures 5B**, **S7A**). NK cells were identified by cytolysis-related enzymes (e.g., *Gzma*, *Gzmb*, *Prf1*) and receptors (e.g., *Klre1*, *Klrb1c*), and BC was marked with early B-associated genes (e.g., *Cd79a*, *Igkc*, and *Ebf1*) (**Figure 5B**). Furthermore, the cytotoxic markers (e.g., *Cd8a*, *Cd8b1*, *Nkg7*) were expressed in TC subtypes (TC1 and TC2), whereas the helper marker (*Cd4*) exhibited low expression (**Figure S7A**). TC2 was activated and entered cell cycling as indicated by proliferative markers (e.g., *Stmn1*, *Mki67*, and *Pclaf*) (**Figure 5B**). UCell analysis showed that TC1 was enriched with naive T genes, while NK or TC2 cells were associated with cytotoxic effectors (**Figure 5C**). The pseudobulk analysis indicated that antigen processing genes (e.g., *H2-Ab1*, *Cd74*) were upregulated (fold change>2, *padj*<0.05) in NK cells of allografts, whereas there was no significant difference in gene expression of TCs or BCs as compared with syngrafts (*padj*>0.05) (**File S2**).

Monocle analysis was performed to assess how cytotoxic TCs were differentiated. By defining TC1 as the differentiation starter due to the enriched naïve gene signature (**Figure 5C**), the state-2 was found as the pseudtime root that converted toward state-1 and 3 from branch point-1 in the trajectory plot (**Figures 5D, E**). Splitting differentiation trajectories also showed that the cell number of TC1 or TC2 was higher in allografts than in syngrafts (**Figure 5F**). The state-3 was the major destiny of TC1, while TC2 was mainly distributed to the state-1 with high *Cd8a* expression (**Figure 5G**). The genes controlling cell fate decisions from branch-1 were shown in a heatmap with GO analysis (**Figure 5H**). The representative genes (which are related to cell cycle or immune response) exhibited an increasing trend in state-1 but were downregulated along state-3 (**Figure 5I**). In contrast, the representative genes (which are related to inflammatory or IL-1 responses) were upregulated in state-3 but downregulated towards state-1 (**Figure 5J**), indicating the dynamic shift in gene expression patterns during TC activation between cell states.

Additionally, the profiling of TCR or BCR repertoire was analyzed by the 5’v1 assay to determine the activation of lymphocytes in response to allogeneic antigens (**Figure S1D**). Totally 303 TCs (48 in syngrafts *vs.* 255 in allografts) and 105 BCs (27 in syngrafts *vs.* 78 in allografts) were recovered after scRNAseq. The Cell Ranger V(D)J pipeline showed that the clonal expansions of TCR or BCR were present in allografts, while only one clone (singleton) was formed in syngrafts (**Figures 5K, L**; **File S3**). Moreover, the percentages of VJ gene recombinations differed significantly between the TCs or BCs in the syngrafts and allografts (**Figures S7B, C**). Although only a few clonotypes were detected, the distinct expression patterns of TCRs and BCRs between syngrafts and allografts suggested an early onset of adaptive immune responses.

### 3.7 Interaction between innate immune cells and T cells

**Figure 6:**
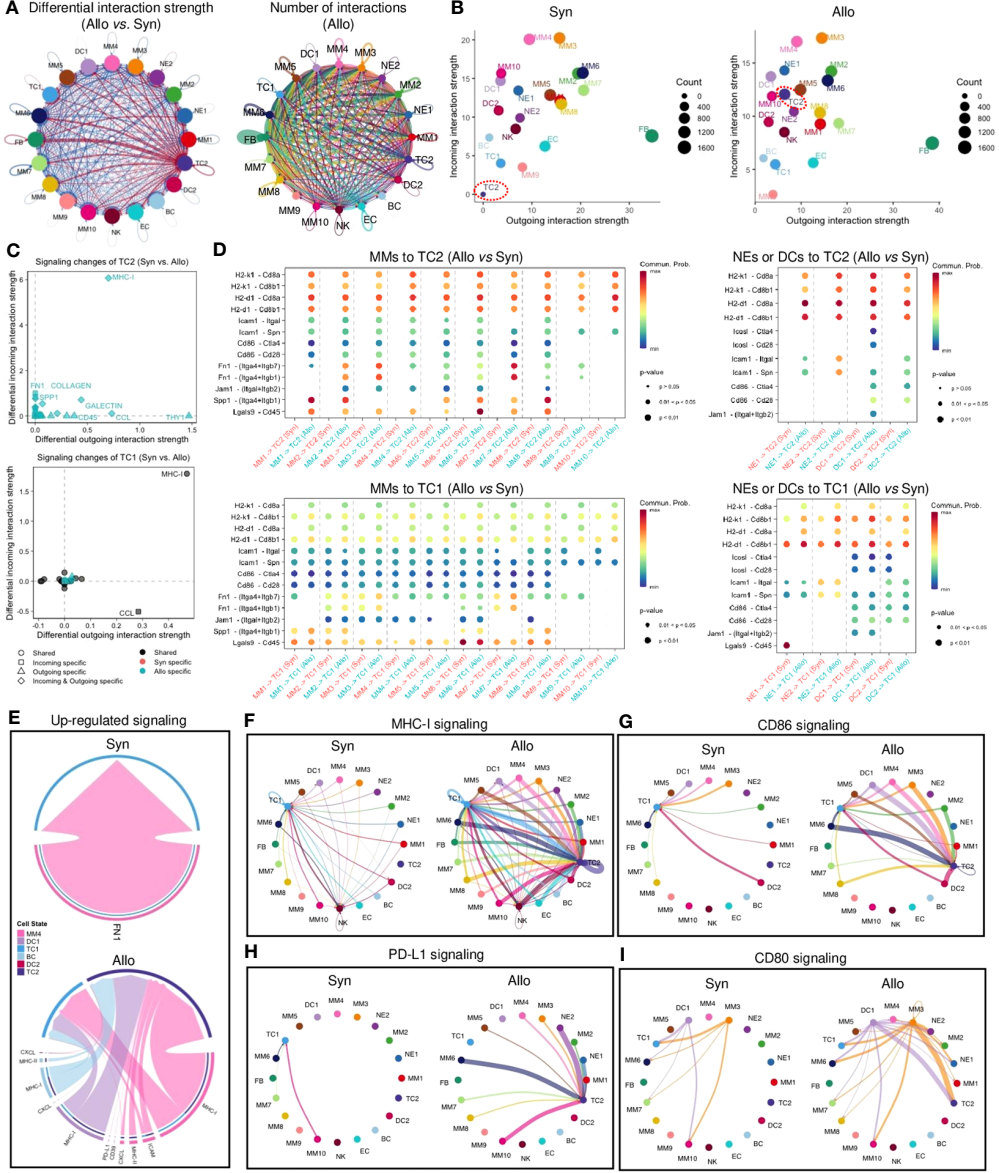
Cell-cell communication network of infiltrating cells. **(A)** Left panel: circle plot showing the differential interaction strength in the cell-cell communication network between syngraft and allograft datasets. Red-colored edges represent increased signaling and blue-colored edges represent decreased signaling in allografts as compared with syngrafts. Right panel: circle plot showing the number of cell-cell interactions (including autocrine and paracrine) in allografts. Circle color is coded according to the cell type and line color indicates the cell source as the sender. Connected line indicates autocrine or paracrine signaling. Thicker line represents more number of interactions. **(B)** Scatter plots showing the overall incoming and outgoing interaction strengths of each cell type in syngrafts or allografts. Dot color is coded according to the cell type. Dot size represents the interaction number shown on the right of each chart. TC2 subset is highlighted by a red dot circle. **(C)** Scatter plots showing the incoming/outgoing signaling strength changes of two T cell types in allografts as compared with syngrafts. The x or y-axis represents the log2 fold change of signaling strength between datasets. The statistical significance is shown by dot color (specific to the sample source) and dot shape (specific to the signaling source). **(D)** Bubble plot showing significantly differential ligand-receptor pairs between allografts and syngrafts, which contribute to the signaling sending from monocytes, macrophages, neutrophils, or dendritic cells to T cells. The dot color and size represent the calculated communication probability and p-values (one-sided permutation test in CellChat), respectively. Empty means no communication (probability is zero). **(E)** Chord diagrams showing upregulated signaling of ligand-receptor pairs sending from antigen-presenting cells (MM4, DC1, DC2, and BC) to T cells in syngrafts or allografts. Color is coded according to the cell type. Edge colors are consistent with the sources as sender. The arrowhead indicates the targets (the inner thinner bars) that receive the signal from the corresponding outer bars. The inner bar size is proportional to the signal strength received by the targets. **(F-I)** Circle plots showing the inferred intercellular communication network of T cell activation-related signaling pathways in syngrafts or allografts. Circle sizes are proportional to the number of cells in each cell group and edge width represents the communication probability.

The CellChat (41) analysis showed the total number of potential interactions (including autocrine and paracrine) between different cell types (**Figures 6A**, **S8A**). The strength of cell interactions was increased in allografts when compared to syngrafts (**Figures 6A**, **S8A**). Overall pathway information showed that interplays involving TC2, either with other cells or with themselves, were markedly altered in allografts when compared to syngrafts (**Figure 6B**). Comparing the differences in the signaling strengths between allografts and syngrafts, the MHC-I pathway was the most significantly changed in TC2 but not in the counterpart TC1 (**Figure 6C**). In addition, distinct pathways specific to allografts (e.g., MHC-II, LCK, ALCAM, and IFN-II) or syngrafts (e.g., SEMA6 and AGRN) were identified by analyzing the overall flow (both incoming and outgoing) within inferred networks (**Figure S8B**). Comparing overall signaling patterns of all cell types also revealed that TC2 in allografts, but not syngrafts, displayed notable activation of innate immune pathways (**Figure S8C**). Thus, interactions between innate immune cells were further dissected to elucidate the mechanism of TC activation.

Analysis of specific ligand-receptor pairs between myeloid cells and TCs revealed that TC2 was the primary responder to stimulation signals (such as H2-k1, H2-d1, Icam1, and Lgals9) in allografts, but not in syngrafts, while TC1 showed no such difference between grafts (**Figure 6D**). MHC-I was one of the upregulated pathways in MMs targeting TCs in allografts but was not changed in syngrafts (**Figures 6E**, **S8D**), while the CCL and CXCL chemotaxis pathways activated in both grafts could be involved in a reparative response for surgical traumatic injuries (42). Mapping of all identified cell types revealed constitutive expression of MHC-I and other co-stimulatory signals such as CD80, CD86, and PD-L1 in several MM or DC subtypes in both syngrafts and allografts (**Figures 6F–I**), suggesting the development of self-awareness in TCs (43). However, these pathways targeting TC2 were more pronounced in allografts compared to syngrafts. The reparative pathways such as FN1 and TGFβ were robustly activated in syngrafts, while their signaling strengths were slightly decreased in allografts (**Figures S8B**, **E**). Thus, identifying potential therapeutic targets in these pathways may reduce TC activation and mitigate acute rejection.

### 3.8 Interferon signaling modulates inflammatory genes in infiltrating monocytes

**Figure 7:**
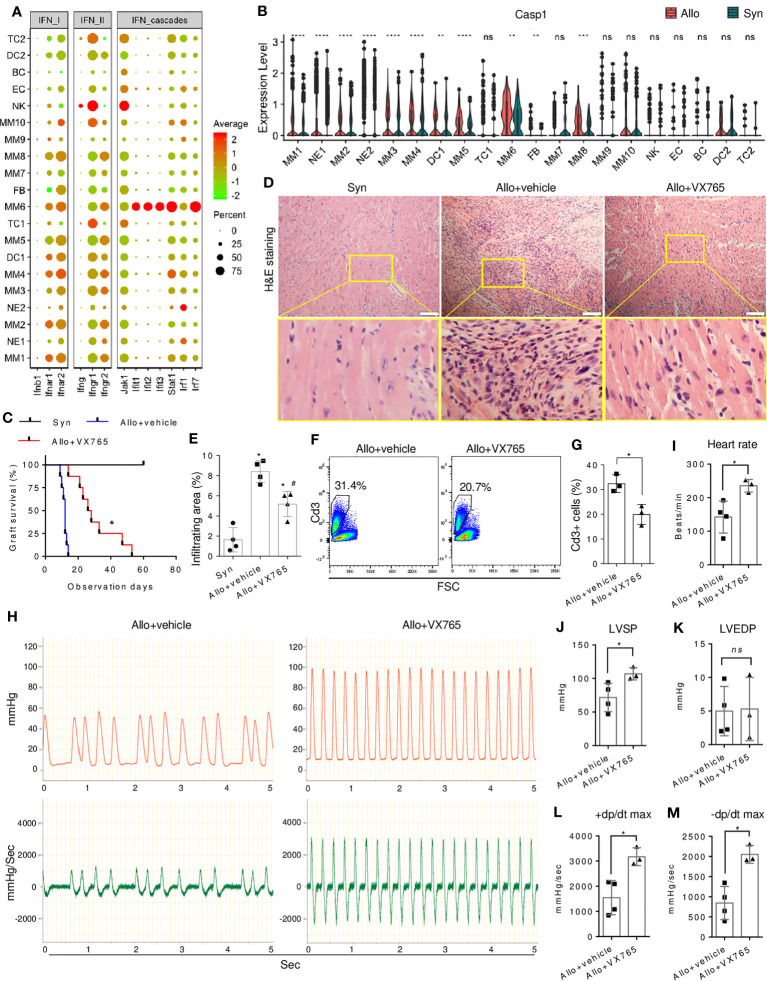
Effect of caspase-1 inhibitor on allograft rejection. **(A)** A dot plot showing the expressions of IFN signaling genes in all cell clusters. The scale of dot size representing cell percentage and dot color representing the average expression is shown on the right. **(B)** Violin plot showing the differential expression level of *Casp1* in the infiltrating cells of allografts as compared with syngrafts. ^ns^ not significant, ** *p*<0.01, *** *p*<0.001, **** *p*<0.0001 (Student’s t-test). **(C)** Survival curves of syngrafts (median survival time > 60 days) and allografts with or without VX765 treatment (27 days *vs.* 12 days). VX765 (50mg/kg) was administrated for one week after the surgery. The complete cessation of a heartbeat is the endpoint. n=8 per group. * *vs* allograft+vehicle, *p*<0.001 (Gehan-Breslow-Wilcoxon test). **(D)** Representative H&E staining of syngrafts and allografts with or without VX765 treatment at one-week post-transplant. Scale bars indicate 50 µm. **(E)** Quantification of immune infiltrating areas corresponding to **(D)**. n=4 per group. * *vs* syngrafts, *p*<0.01; ^#^ *vs* allograft+vehicle, *p*<0.01 (one-way ANOVA with Bonferroni’s multiple comparisons test). **(F)** Flow cytometry showing percentages of Cd3^+^ cells in the cells dissociated from allografts with or without VX765 treatment at one-week post-transplant. **(G)** Quantification of percentages of Cd3^+^ cells corresponding to **(F)**. n=3 per group. * *p*<0.05 (Student’s t-test). **(H)** Representative beating images of allografts with or without VX765 treatment at one-week post-transplant. The red wave represents the left ventricle pressure (mmHg). The green wave represents the left ventricle pressure per sec ± dp/dt (mmHg/s). **(I–M)** Heart rate, left ventricle systolic pressure (LVSP), left ventricle end-diastolic pressure (LVEDP), maximum dp/dt in the systolic period, and maximum dp/dt in the diastolic period of allografts with or without VX765 treatment. The first nine waves of each sample were measured. n=3~4 per group. ^ns^ not significant, * *p* < 0.05 (Student’s t-test).

The above data suggested the potential role of pro-inflammatory IFN signaling in innate immunity and allograft rejection. However, the specific signaling molecule that can be targeted for drug intervention remains unknown. During the initial phase of transplantation, NK cells were identified as the primary source of IFN-γ signaling that targeted MMs or DCs (**Figures 7A**, **S8F**), while the expression of IFN-I cytokines (such as IFN-β) remained minimal within the infiltrated cells. Further investigation of signaling effectors in targeted cells is warranted due to the pleiotropic nature of IFN-γ and its dual role in immune rejection or tolerance (44). Despite the ubiquitous expression of IFN-γ receptors in immune cells, MM6 was a major responder to IFN-γ signaling (**Figures 7A**, **S9A**). IFN-γ downstream TFs such as *Stat1* and *Irf1* were upregulated in MM6 of allografts (**Figure 2F**), accompanied by high expression of downstream genes such as MHC and other molecules (**Figure S9A**). However, treatment with the chemical Stat1 inhibitor (Fludarabine (45)) did not improve heart graft survival, as evidenced by no significant difference in the median survival time of allografts (**Figure S9B**). IFN-γ could activate innate immune cells through crosstalk with alternative effectors.

The differential expression and regulon analysis of MMs revealed the involvement of proinflammatory factors such as *Casp1* (**Figures 2D, G**), which is associated with IFN signaling (46) and further piqued our interest. Caspase-1 is a critical component of the NLRP3 inflammasome that regulates cardiac remodeling post-infarction (47), whereas its role in rejection is unknown. Inflammasome components were differentially expressed in infiltrates, but not activated in non-myeloid cells (**Figure S9C**), with MM6 highly expressing *Casp1* and *Il18*, and NE2 expressing *Nlrp3* and *Il1b*. Furthermore, allograft-infiltrating myeloid cells, particularly the MM6 population, exhibited up-regulation of *Casp1* as compared with syngrafts (**Figure 7B**). MM6 can interact with TC2 (**Figures 6D–H**) or other myeloid subtypes (**Figure S9D**). In allografts, Ccl8 signaling of MM6 targeted Ccr1 and Ccr2 which is known to induce immune cell recruitment and migration, while this signaling was absent in syngrafts. Moreover, MM6 selectively interacted with other subtypes in allografts through the interplay between Lair1 and Pira2 which is crucial for alloantigen-specific memory of innate myeloid cells (48). Therefore, MM6 cells were the primary monocyte population involved in inflammasome activation and closely associated with immune infiltration and alloimmunity initiation.

### 3.9 Inhibition of caspase-1 delays acute rejection

Activation of inflammasome-related components, including caspase-1, was observed in allografts rather than syngrafts within the first 7 days post-transplant (**Figure S9E**), as confirmed by immunoblotting and consistent with our scRNAseq analysis. Subsequently, we tested whether VX765 (a caspase-1 inhibitor) (49) can improve heart graft survival and function. The median survival time of allografts was significantly increased by VX765 (**Figure 7C**). Cell infiltration was decreased in allografts by VX765 as compared with the vehicle control (**Figures 7D, E**). The VX765-treated heart allografts showed improved structural remodeling and alleviated inter- and intracellular edema. The percentage of infiltrating CD3^+^ TCs was reduced in allografts by VX765 (**Figures 7F, G**). In addition, the graft ventricular pressure or contractility was detected after restoring the sinus rhythm. The pressure peak and pressure-changed-rate peak of allografts treated with VX765 were higher than that of allografts with the vehicle control (**Figure 7H**). The ventricular systolic pressure and heart rate of allografts were increased by the VX765 treatment as compared with the vehicle control (**Figures 7I–M**), while there was no significant difference in the ventricular end-diastolic pressures. The +dp/dt max and the -dp/dt max of allografts were rapidly raised by VX765, reflecting an improved contractile function.

## 4 Discussion

Cell infiltration in response to surgical trauma and/or ischemia/reperfusion injuries is a conserved and widely acknowledged phenomenon in clinical transplantation (50, 51). The immune rejection involves intricate interactions between host cells that infiltrate the graft in response to non-self antigens (52). This study of scRNAseq analysis provided profound insights into the cell landscape of recipient-derived infiltrates in heart grafts during the early immune response following transplantation. Our findings identified 18 clusters of immune cells and 2 clusters of non-immune cells, revealing new insights into components of graft-infiltrating cells.

We found a substantial influx of MMs as a key characteristic of the early stages of heart transplantation. Although the MM identities were similar between the syngrafts and allografts, the phenotypes associated with IFN-γ signaling, inflammation, or antigen processing were more prevalent in the allografts. Notably, monocytes exhibit cell plasticity, and their activation state can be influenced by their microenvironment (53). Our scRNAseq analysis also showed the *Ly6c2*/*Ccr2*-expressing monocytes as the root cells for macrophage differentiation, revealing sequential stages of metabolism, cell size, and cytoplasmic functional maturation, which was consistent with previous studies of innate immune responses in transplant rejection (51, 54). While the differentiation direction of MMs was similar in syngrafts and allografts, allografts showed a higher proportion of MM subsets (e.g. MM4) that transitioned to cellular states associated with antigen processing and T-cell activation. Differentiation of monocytes into mature APCs that recruit in allografts and recognize non-self signals can initiate transplant rejection (55, 56). Therefore, the identified cell subsets exhibiting both macrophage and APC gene signatures played a critical role in the early-stage allograft rejection response. Interestingly, pro-inflammatory factors such as IFN-γ can shift monocyte differentiation to macrophages rather than DCs (57), while it remains unknown whether this mechanism can be recapitulated in an *in vivo* setting of heart transplantation.

Other myeloid cells including DCs and NEs were observed to migrate to both syngrafts and allografts. The gene marker profiles showed that the infiltrating DCs and NEs resembled cDCs and pro-inflammatory neutrophils, respectively. cDC1 cells are known to prime and activate CD8^+^ TCs, while cDC2 cells are more efficient at activating CD4^+^ TCs and promoting humoral immunity (58). NE activation and recruitment can initiate an inflammatory response and stimulate other immune cells, which can result in tissue damage and ultimately rejection of the transplant (59). Although the proportions of NEs or DCs were comparable in allografts and syngrafts during the early transplant phase, pseudobulk analysis of significant genes revealed increased innate immune responses such as IFN-β signaling in allografts. IFN-β plays a crucial role in mediating the expression of MHC or co-stimulatory molecules in DCs, facilitating antigen processing and presentation (60). Additionally, IFN-β serves as a key regulator in neutrophil activation, encompassing functions such as tissue recruitment and chemokine production (61). While IFN-β is known as a pro-inflammatory factor, it demonstrates a paradoxical effect by extending allograft survival through promoting regulatory TC induction, which is mediated by Stat1- and P300-dependent Foxp3 acetylation (62). Thus, further research is required to gain a comprehensive understanding of the specific impact of IFN-β on DCs or NEs during the initiation of alloimmunity following heart transplantation.

In addition, our study identified a small population of infiltrating lymphocytes (NK cells, TCs, and BCs) during the early transplant phase. Similar gene profiles of TCs or BCs in syngrafts and allografts suggested that early involvement of lymphocytes in heart transplants was not antigen-specific. Instead, it may be induced by the inflammatory milieu or the release of damage-associated molecular patterns (63). Notably, the allografts exhibited elevated proportions of cytotoxic TCs and NK cells, accompanied by enhanced cellular proliferation and TCR/BCR clonal expansion. Cell-cell communication analysis also showed that the infiltrating mononuclear phagocytes provided strong signals of MHC-I and co-stimulatory molecules to target the proliferating TCs in allografts. A key insight from these findings is the confirmation of the pivotal role played by the indirect pathway of allorecognition, whereby graft-infiltrating host cells interact and contribute significantly to the initiation of the alloimmune response (64). Therefore, the TC activation and initiation of the adaptive immune response during the early transplant phase are contingent upon the recipient’s innate immune signaling. Targeting this signaling can mitigate the incidence of allograft rejection while avoiding adverse outcomes associated with systemic TC depletion (65, 66).

Our scRNAseq analysis revealed a robust association between the alloimmune response and the expression of inflammation-related genes in graft-infiltrating cells. Cell apoptosis-induced activation of inflammasomes is associated with cardiac allograft rejection (67). Caspase-1 activation is triggered by the assembly of inflammasome complexes, particularly Nlrp3, which function as sensors for ‘danger’ molecules or non-self signals, such as donor-derived cell-free DNA, in the context of organ transplantation (55, 68, 69). Caspase-1 leads to pyroptosis, a process of programmed cell death that includes cell swelling, membrane rupture, and release of cytosolic contents (70). The further release of adhesion molecules and chemokines can lead to neutrophil sequestration, monocyte recruitment, and TC activation, all of which are key steps in the continuum from allograft insult to chronic dysfunction (71). The development of drugs targeting inflammasomes aids in inducing transplant tolerance without complete immunoparalysis (72). We observed high expression of caspase-1 in allograft-infiltrating monocyte subsets, and inhibition of caspase-1 resulted in improved contractility function, decreased immune infiltration after transplant, and sustained high survival rates for over 4 weeks. Our study is the first to investigate the expression of inflammasome components at a single-cell level in heart grafts, which provides valuable information on the cellular and molecular mechanisms underlying cardiac allograft rejection and the potential targets (such as using specific caspase-1 inhibitors) for therapeutic intervention. However, further investigation is needed to assess the optimization of treatment strategies, the temporal dynamics of the inflammasome pathway, and the role of caspase-1 in long-term or chronic rejection. Additionally, comprehensive research is required to elucidate the mechanisms underlying caspase-1 expression induction and its intricate interplay with other inflammation-related pathways in the context of heart allograft rejection.

In summary, we characterized mouse recipient-derived infiltrates and immune responses in heart grafts at the early stage of transplantation. The pro-inflammatory pathways were identified by comparing the differentially expressed genes between syngrafts and allografts. Cell-cell communications among the graft-infiltrating cells showed the potential of indirect allorecognition signaling. The scRNAseq dataset also revealed several potential targets such as caspase-1 that can be assessed to develop new strategies for reducing immune rejection and improving graft function.

## Data availability statement

The datasets presented in this study can be found in online repositories. The names of the repository/repositories and accession number(s) can be found below: CRA007855 (GSA, https://bigd.big.ac.cn/gsa/browse/CRA007855).

## Ethics statement

The animal study was approved by Medical Ethics Committee of Guangdong Provincial People’s Hospital, Guangdong Academy of Medical Sciences. The study was conducted in accordance with the local legislation and institutional requirements.

## Author contributions

ZW and JL: Conception and experiment design, performed animal surgery, collection of samples, assessed graft function, assembly of data, bioinformatics analysis and interpretation, and manuscript writing. SZ: Experiment design, assembly of data, performed data analysis, data interpretation, and manuscript writing. NL, MZ, FX, GL, CY, and CJ: Performed data analysis, provided materials or reagents, and assisted with animal experiments. JM, TS, and PZ: Surprised experiments, design, financial support, comments, discussion, and manuscript editing. All authors contributed to the article and approved the submitted version.

## References

[1] Guglin M Zucker MJ Borlaug BA Breen E Cleveland J Johnson MR. Evaluation for heart transplantation and LVAD implantation: JACC council perspectives. J Am Coll Cardiol (2020) 75(12):1471–87. doi: 10.1016/j.jacc.2020.01.034 32216916

[2] Madsen JC. Advances in the immunology of heart transplantation. J Heart Lung Transplant (2017) 36(12):1299–305. doi: 10.1016/j.healun.2017.10.003 PMC5895083 29173391

[3] Lopez-Sainz A Barge-Caballero E Barge-Caballero G Couto-Mallon D Paniagua-Martin MJ Seoane-Quiroga L. Late graft failure in heart transplant recipients: incidence, risk factors and clinical outcomes. Eur J Heart Fail (2018) 20(2):385–94. doi: 10.1002/ejhf.886 28580728

[4] Iwasaki A Medzhitov R. Control of adaptive immunity by the innate immune system. Nat Immunol (2015) 16(4):343–53. doi: 10.1038/ni.3123 PMC4507498 25789684

[5] Spahn JH Li W Kreisel D. Innate immune cells in transplantation. Curr Opin Organ Transplant (2014) 19(1):14–9. doi: 10.1097/MOT.0000000000000041 PMC4285410 24316757

[6] Christen T Nahrendorf M Wildgruber M Swirski FK Aikawa E Waterman P. Molecular imaging of innate immune cell function in transplant rejection. Circulation (2009) 119(14):1925–32. doi: 10.1161/CIRCULATIONAHA.108.796888 PMC2676889 19332470

[7] Zhuang Q Liu Q Divito SJ Zeng Q Yatim KM Hughes AD. Graft-infiltrating host dendritic cells play a key role in organ transplant rejection. Nat Commun (2016) 7(1):12623. doi: 10.1038/ncomms12623 27554168 PMC4999515

[8] DeWolf S Sykes M. Alloimmune T cells in transplantation. J Clin Invest (2017) 127(7):2473–81. doi: 10.1172/JCI90595 PMC5490749 28628037

[9] LaRosa DF Rahman AH Turka LA. The innate immune system in allograft rejection and tolerance. J Immunol (2007) 178(12):7503–9. doi: 10.4049/jimmunol.178.12.7503 PMC2840045 17548582

[10] Martini E Kunderfranco P Peano C Carullo P Cremonesi M Schorn T. Single-cell sequencing of mouse heart immune infiltrate in pressure overload-driven heart failure reveals extent of immune activation. Circulation (2019) 140(25):2089–107. doi: 10.1161/CIRCULATIONAHA.119.041694 31661975

[11] Hua X Hu G Hu Q Chang Y Hu Y Gao L. Single-cell RNA sequencing to dissect the immunological network of autoimmune myocarditis. Circulation (2020) 142(4):384–400. doi: 10.1161/CIRCULATIONAHA.119.043545 32431172

[12] Kopecky BJ Dun H Amrute JM Lin CY Bredemeyer AL Terada Y. Donor macrophages modulate rejection after heart transplantation. Circulation (2022) 146(8):623–38. doi: 10.1161/CIRCULATIONAHA.121.057400 PMC9398940 35880523

[13] Chang Y Li X Cheng Q Hu Y Chen X Hua X. Single-cell transcriptomic identified HIF1A as a target for attenuating acute rejection after heart transplantation. Basic Res Cardiol (2021) 116(1):64. doi: 10.1007/s00395-021-00904-5 34870762

[14] Kong D Huang S Miao X Li J Wu Z Shi Y. The dynamic cellular landscape of grafts with acute rejection after heart transplantation. J Heart Lung Transplant (2023) 42(2):160–72. doi: 10.1016/j.healun.2022.10.017 36411190

[15] Tang Y Wang J Zhang Y Li J Chen M Gao Y. Single-cell RNA sequencing identifies intra-graft population heterogeneity in acute heart allograft rejection in mouse. Front Immunol (2022) 13:832573. doi: 10.3389/fimmu.2022.832573 35222420 PMC8866760

[16] Chen Z Xu H Li Y Zhang X Cui J Zou Y. Single-Cell RNA sequencing reveals immune cell dynamics and local intercellular communication in acute murine cardiac allograft rejection. Theranostics (2022) 12(14):6242–57. doi: 10.7150/thno.75543 PMC9475451 36168621

[17] Plenter RJ Grazia TJ. Murine heterotopic heart transplant technique. J Vis Exp (2014) 89:e51511. doi: 10.3791/51511 PMC4212889 25046118

[18] Aronoff L Epelman S Clemente-Casares X. Isolation and identification of extravascular immune cells of the heart. J Vis Exp (2018) 138:58114. doi: 10.3791/58114 PMC6231712 30199044

[19] Satija R Farrell JA Gennert D Schier AF Regev A. Spatial reconstruction of single-cell gene expression data. Nat Biotechnol (2015) 33(5):495–502. doi: 10.1038/nbt.3192 25867923 PMC4430369

[20] McGinnis CS Murrow LM Gartner ZJ. DoubletFinder: doublet detection in single-cell RNA sequencing data using artificial nearest neighbors. Cell Syst (2019) 8(4):329–337 e4. doi: 10.1016/j.cels.2019.03.003 30954475 PMC6853612

[21] Aran D Looney AP Liu L Wu E Fong V Hsu A. Reference-based analysis of lung single-cell sequencing reveals a transitional profibrotic macrophage. Nat Immunol (2019) 20(2):163–72. doi: 10.1038/s41590-018-0276-y PMC6340744 30643263

[22] Andreatta M Carmona SJ. UCell: Robust and scalable single-cell gene signature scoring. Comput Struct Biotechnol J (2021) 19:3796–8. doi: 10.1016/j.csbj.2021.06.043 PMC8271111 34285779

[23] Murray PJ Allen JE Biswas SK Fisher EA Gilroy DW Goerdt S. Macrophage activation and polarization: nomenclature and experimental guidelines. Immunity (2014) 41(1):14–20. doi: 10.1016/j.immuni.2014.06.008 25035950 PMC4123412

[24] Love MI Huber W Anders S. Moderated estimation of fold change and dispersion for RNA-seq data with DESeq2. Genome Biol (2014) 15(12):550. doi: 10.1186/s13059-014-0550-8 25516281 PMC4302049

[25] Aibar S Gonzalez-Blas CB Moerman T Huynh-Thu VA Imrichova H Hulselmans G. : SCENIC: single-cell regulatory network inference and clustering. Nat Methods (2017) 14(11):1083–6. doi: 10.1038/nmeth.4463 PMC5937676 28991892

[26] Suo S Zhu Q Saadatpour A Fei L Guo G Yuan GC. Revealing the critical regulators of cell identity in the mouse cell atlas. Cell Rep (2018) 25(6):1436–1445 e3. doi: 10.1016/j.celrep.2018.10.045 30404000 PMC6281296

[27] Langlais D Barreiro LB Gros P. The macrophage IRF8/IRF1 regulome is required for protection against infections and is associated with chronic inflammation. J Exp Med (2016) 213(4):585–603. doi: 10.1084/jem.20151764 27001747 PMC4821649

[28] Kim B-H Shenoy AR Kumar P Das R Tiwari S MacMicking JD. A family of IFN-γ–inducible 65-kD GTPases protects against bacterial infection. Science (2011) 332(6030):717–21. doi: 10.1126/science.1201711 21551061

[29] Honda K Yanai H Negishi H Asagiri M Sato M Mizutani T. IRF-7 is the master regulator of type-I interferon-dependent immune responses. Nature (2005) 434(7034):772–7. doi: 10.1038/nature03464 15800576

[30] Platanias LC. Mechanisms of type-I- and type-II-interferon-mediated signalling. Nat Rev Immunol (2005) 5(5):375–86. doi: 10.1038/nri1604 15864272

[31] Riess M Fuchs NV Idica A Hamdorf M Flory E Pedersen IM. Interferons Induce Expression of SAMHD1 in Monocytes through Down-regulation of miR-181a and miR-30a. J Biol Chem (2017) 292(1):264–77. doi: 10.1074/jbc.M116.752584 PMC5217685 27909056

[32] Trapnell C Cacchiarelli D Grimsby J Pokharel P Li S Morse M. The dynamics and regulators of cell fate decisions are revealed by pseudotemporal ordering of single cells. Nat Biotechnol (2014) 32(4):381–6. doi: 10.1038/nbt.2859 PMC4122333 24658644

[33] Ripoll VM Irvine KM Ravasi T Sweet MJ Hume DA. Gpnmb is induced in macrophages by IFN-gamma and lipopolysaccharide and acts as a feedback regulator of proinflammatory responses. J Immunol (2007) 178(10):6557–66. doi: 10.4049/jimmunol.178.10.6557 17475886

[34] Guilliams M Ginhoux F Jakubzick C Naik SH Onai N Schraml BU. Dendritic cells, monocytes and macrophages: a unified nomenclature based on ontogeny. Nat Rev Immunol (2014) 14(8):571–8. doi: 10.1038/nri3712 PMC4638219 25033907

[35] Crinier A Milpied P Escaliere B Piperoglou C Galluso J Balsamo A. High-dimensional single-cell analysis identifies organ-specific signatures and conserved NK cell subsets in humans and mice. Immunity (2018) 49(5):971–986 e5. doi: 10.1016/j.immuni.2018.09.009 30413361 PMC6269138

[36] Whittaker Hawkins RF Patenaude A Dumas A Jain R Tesfagiorgis Y Kerfoot S. ICAM1+ neutrophils promote chronic inflammation *via* ASPRV1 in B cell-dependent autoimmune encephalomyelitis. JCI Insight (2017) 2(23):e96882. doi: 10.1172/jci.insight.96882 29212956 PMC5752297

[37] Calcagno DM Zhang C Toomu A Huang K Ninh VK Miyamoto S. SiglecF(HI) marks late-stage neutrophils of the infarcted heart: A single-cell transcriptomic analysis of neutrophil diversification. J Am Heart Assoc (2021) 10(4):e019019. doi: 10.1161/jaha.120.019019 33525909 PMC7955351

[38] McGeough MD Wree A Inzaugarat ME Haimovich A Johnson CD Pena CA. TNF regulates transcription of NLRP3 inflammasome components and inflammatory molecules in cryopyrinopathies. J Clin Invest (2017) 127(12):4488–97. doi: 10.1172/JCI90699 PMC5707143 29130929

[39] Fridlender ZG Sun J Kim S Kapoor V Cheng G Ling L. Polarization of tumor-associated neutrophil phenotype by TGF-beta: "N1" versus "N2" TAN. Cancer Cell (2009) 16(3):183–94. doi: 10.1016/j.ccr.2009.06.017 PMC2754404 19732719

[40] Garcia-Culebras A Duran-Laforet V Pena-Martinez C Moraga A Ballesteros I Cuartero MI. Role of TLR4 (Toll-like receptor 4) in N1/N2 neutrophil programming after stroke. Stroke (2019) 50(10):2922–32. doi: 10.1161/STROKEAHA.119.025085 31451099

[41] Jin S Guerrero-Juarez CF Zhang L Chang I Ramos R Kuan CH. Inference and analysis of cell-cell communication using CellChat. Nat Commun (2021) 12(1):1088. doi: 10.1038/s41467-021-21246-9 33597522 PMC7889871

[42] Noels H Weber C Koenen RR. Chemokines as therapeutic targets in cardiovascular disease. Arterioscler Thromb Vasc Biol (2019) 39(4):583–92. doi: 10.1161/ATVBAHA.118.312037 30760014

[43] Malissen B Bongrand P. Early T cell activation: integrating biochemical, structural, and biophysical cues. Annu Rev Immunol (2015) 33:539–61. doi: 10.1146/annurev-immunol-032414-112158 25861978

[44] Rozman P Svajger U. The tolerogenic role of IFN-gamma. Cytokine Growth Factor Rev (2018) 41:40–53. doi: 10.1016/j.cytogfr.2018.04.001 29655565

[45] Torella D Curcio A Gasparri C Galuppo V De Serio D Surace FC. Fludarabine prevents smooth muscle proliferation in *vitro* and neointimal hyperplasia in *vivo* through specific inhibition of STAT-1 activation. Am J Physiol Heart Circ Physiol (2007) 292(6):H2935–43. doi: 10.1152/ajpheart.00887.2006 17293493

[46] Kopitar-Jerala N. The role of interferons in inflammation and inflammasome activation. Front Immunol (2017) 8:873. doi: 10.3389/fimmu.2017.00873 28791024 PMC5525294

[47] Toldo S Abbate A. The NLRP3 inflammasome in acute myocardial infarction. Nat Rev Cardiol (2018) 15(4):203–14. doi: 10.1038/nrcardio.2017.161 29143812

[48] Dai H Lan P Zhao D Abou-Daya K Liu W Chen W. PIRs mediate innate myeloid cell memory to nonself MHC molecules. Science (2020) 368(6495):1122–7. doi: 10.1126/science.aax4040 PMC7379379 32381589

[49] Audia JP Yang XM Crockett ES Housley N Haq EU O'Donnell K. Caspase-1 inhibition by VX-765 administered at reperfusion in P2Y(12) receptor antagonist-treated rats provides long-term reduction in myocardial infarct size and preservation of ventricular function. Basic Res Cardiol (2018) 113(5):32. doi: 10.1007/s00395-018-0692-z 29992382 PMC6396295

[50] Luster AD Alon R von Andrian UH. Immune cell migration in inflammation: present and future therapeutic targets. Nat Immunol (2005) 6(12):1182–90. doi: 10.1038/ni1275 16369557

[51] Ochando J Ordikhani F Boros P Jordan S. The innate immune response to allotransplants: mechanisms and therapeutic potentials. Cell Mol Immunol (2019) 16(4):350–6. doi: 10.1038/s41423-019-0216-2 PMC6462017 30804476

[52] Abou-Daya KI Oberbarnscheidt MH. Innate allorecognition in transplantation. J Heart Lung Transplant (2021) 40(7):557–61. doi: 10.1016/j.healun.2021.03.018 PMC8238827 33958265

[53] Jakubzick CV Randolph GJ Henson PM. Monocyte differentiation and antigen-presenting functions. Nat Rev Immunol (2017) 17(6):349–62. doi: 10.1038/nri.2017.28 28436425

[54] Zhao Y Chen S Lan P Wu C Dou Y Xiao X. Macrophage subpopulations and their impact on chronic allograft rejection versus graft acceptance in a mouse heart transplant model. Am J Transplant (2018) 18(3):604–16. doi: 10.1111/ajt.14543 PMC5820161 29044999

[55] Oberbarnscheidt MH Zeng Q Li Q Dai H Williams AL Shlomchik WD. Non-self recognition by monocytes initiates allograft rejection. J Clin Invest (2014) 124(8):3579–89. doi: 10.1172/JCI74370 PMC4109551 24983319

[56] Chow KV Delconte RB Huntington ND Tarlinton DM Sutherland RM Zhan Y. Innate allorecognition results in rapid accumulation of monocyte-derived dendritic cells. J Immunol (2016) 197(5):2000–8. doi: 10.4049/jimmunol.1600181 27474076

[57] Delneste Y Charbonnier P Herbault N Magistrelli G Caron G Bonnefoy JY. Interferon-gamma switches monocyte differentiation from dendritic cells to macrophages. Blood (2003) 101(1):143–50. doi: 10.1182/blood-2002-04-1164 12393446

[58] Schroth S Glinton K Luo X Thorp EB. Innate functions of dendritic cell subsets in cardiac allograft tolerance. Front Immunol (2020) 11:869. doi: 10.3389/fimmu.2020.00869 32431717 PMC7214785

[59] Scozzi D Ibrahim M Menna C Krupnick AS Kreisel D Gelman AE. The role of neutrophils in transplanted organs. Am J Transplant (2017) 17(2):328–35. doi: 10.1111/ajt.13940 PMC5183560 27344051

[60] Simmons DP Wearsch PA Canaday DH Meyerson HJ Liu YC Wang Y. Type I IFN drives a distinctive dendritic cell maturation phenotype that allows continued class II MHC synthesis and antigen processing. J Immunol (2012) 188(7):3116–26. doi: 10.4049/jimmunol.1101313 PMC3311734 22371391

[61] Rocha BC Marques PE Leoratti FMS Junqueira C Pereira DB Antonelli L. Type I interferon transcriptional signature in neutrophils and low-density granulocytes are associated with tissue damage in malaria. Cell Rep (2015) 13(12):2829–41. doi: 10.1016/j.celrep.2015.11.055 PMC4698035 26711347

[62] Fueyo-Gonzalez F McGinty M Ningoo M Anderson L Cantarelli C Andrea A. Interferon-beta acts directly on T cells to prolong allograft survival by enhancing regulatory T cell induction through Foxp3 acetylation. Immunity (2022) 55(3):459–474.e7. doi: 10.1016/j.immuni.2022.01.011 35148827 PMC8917088

[63] Dwyer GK Turnquist HR. Untangling local pro-inflammatory, reparative, and regulatory damage-associated molecular-patterns (DAMPs) pathways to improve transplant outcomes. Front Immunol (2021) 12:611910. doi: 10.3389/fimmu.2021.611910 33708206 PMC7940545

[64] Zhao D Abou-Daya KI Dai H Oberbarnscheidt MH Li XC Lakkis FG. Innate allorecognition and memory in transplantation. Front Immunol (2020) 11:918. doi: 10.3389/fimmu.2020.00918 32547540 PMC7270276

[65] Garrod KR Liu FC Forrest LE Parker I Kang SM Cahalan MD. NK cell patrolling and elimination of donor-derived dendritic cells favor indirect alloreactivity. J Immunol (2010) 184(5):2329–36. doi: 10.4049/jimmunol.0902748 PMC3125519 20139277

[66] van den Bosch TP Kannegieter NM Hesselink DA Baan CC Rowshani AT. Targeting the monocyte-macrophage lineage in solid organ transplantation. Front Immunol (2017) 8:153. doi: 10.3389/fimmu.2017.00153 28261211 PMC5312419

[67] Seto T Kamijo S Wada Y Yamaura K Takahashi K Komatsu K. Upregulation of the apoptosis-related inflammasome in cardiac allograft rejection. J Heart Lung Transplant (2010) 29(3):352–9. doi: 10.1016/j.healun.2009.09.008 20036165

[68] Broz P Dixit VM. Inflammasomes: mechanism of assembly, regulation and signalling. Nat Rev Immunol (2016) 16(7):407–20. doi: 10.1038/nri.2016.58 27291964

[69] Burke RM Dale BL Dholakia S. The NLRP3 inflammasome: relevance in solid organ transplantation. Int J Mol Sci (2021) 22(19):10721. doi: 10.3390/ijms221910721 PMC8509131 34639062

[70] Man SM Kanneganti TD. Regulation of inflammasome activation. Immunol Rev (2015) 265(1):6–21. doi: 10.1111/imr.12296 25879280 PMC4400844

[71] Weigt SS Palchevskiy V Belperio JA. Inflammasomes and IL-1 biology in the pathogenesis of allograft dysfunction. J Clin Invest (2017) 127(6):2022–9. doi: 10.1172/JCI93537 PMC5451233 28569730

[72] Amores-Iniesta J Barbera-Cremades M Martinez CM Pons JA Revilla-Nuin B Martinez-Alarcon L. Extracellular ATP activates the NLRP3 inflammasome and is an early danger signal of skin allograft rejection. Cell Rep (2017) 21(12):3414–26. doi: 10.1016/j.celrep.2017.11.079 PMC5746605 29262323

