# Supplementary material for: Single-cell analysis of graft-infiltrating host cells identifies caspase-1 as a potential therapeutic target for heart transplant rejection
Source: Front Immunol. 2023 Sep 13;14:1251028. doi: 10.3389/fimmu.2023.1251028 (PMC10535112; doi:10.3389/fimmu.2023.1251028)
Supplement: Supplementary file 1 [file DataSheet_1.pdf]

Figure S1

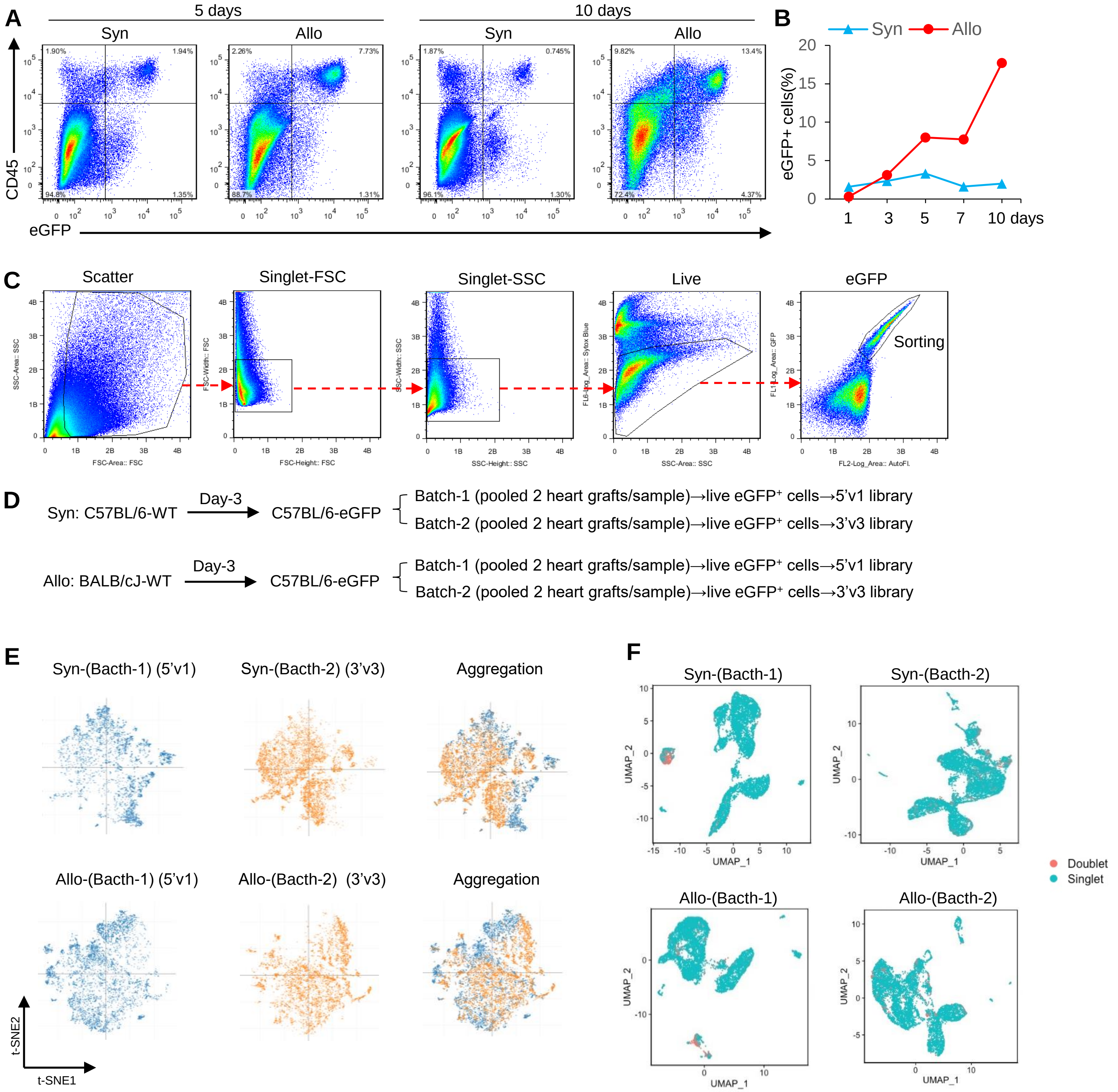

A
5 days
10 days
Syn
Allo
Syn
Allo
CD45
eGFP
1.90%
1.94%
94.8%
1.35%
2.26%
7.73%
88.7%
1.31%
1.87%
0.745%
96.1%
1.30%
9.82%
13.4%
72.4%
4.37%
B
Syn
Allo
eGFP+ cells(%)
1
3
5
7
10 days
C
Scatter
Singlet-FSC
Singlet-SSC
Live
eGFP
Sorting
FSC-Area:: FSC
FSC-Height:: FSC
SSC-Height:: SSC
SSC-Area:: SSC
FL2-Log_Area:: AutoFl.
D
Syn: C57BL/6-WT
Day-3
C57BL/6-eGFP
Batch-1 (pooled 2 heart grafts/sample)→live eGFP+ cells→5'v1 library
Batch-2 (pooled 2 heart grafts/sample)→live eGFP+ cells→3'v3 library
Allo: BALB/cJ-WT
Day-3
C57BL/6-eGFP
Batch-1 (pooled 2 heart grafts/sample)→live eGFP+ cells→5'v1 library
Batch-2 (pooled 2 heart grafts/sample)→live eGFP+ cells→3'v3 library
E
Syn-(Bacth-1) (5'v1)
Syn-(Bacth-2) (3'v3)
Aggregation
Allo-(Bacth-1) (5'v1)
Allo-(Bacth-2) (3'v3)
Aggregation
t-SNE2
t-SNE1
F
Syn-(Bacth-1)
Syn-(Bacth-2)
Allo-(Bacth-1)
Allo-(Bacth-2)
UMAP_1
UMAP_2
Doublet
Singlet

Figure S1. Cell sorting for single-cell RNA sequencing. (A) Representative flow cytometry imaging of the infiltrating $eGFP^+$ or $Cd45^+$ cells in syngrafts or allografts at 5 or 10 days post-transplant. (B) Quantification of percentages of eGFP+ cells corresponding to (A). (C) Fluorescence-activated cell sorting (FACS) of live eGFP cells ($eGFP^+$ and SYTOX™ $Blue^-$) in an allograft. (D) Schematic of experiment design including the mouse groups of heart transplant and library constructions for scRNA-seq. (E) T-distributed stochastic neighbor embedding (TSNE) plots of syngraft or allograft infiltrating cells as viewed by the 10×Loupe Browser after the Cell Ranger analysis. (F) Uniform manifold approximation and projection (UMAP) plots of singlets and doublets in graft-infiltrating cells in each sample after generating the Seurat objects and dimensionality reduction for each library.

Figure S2

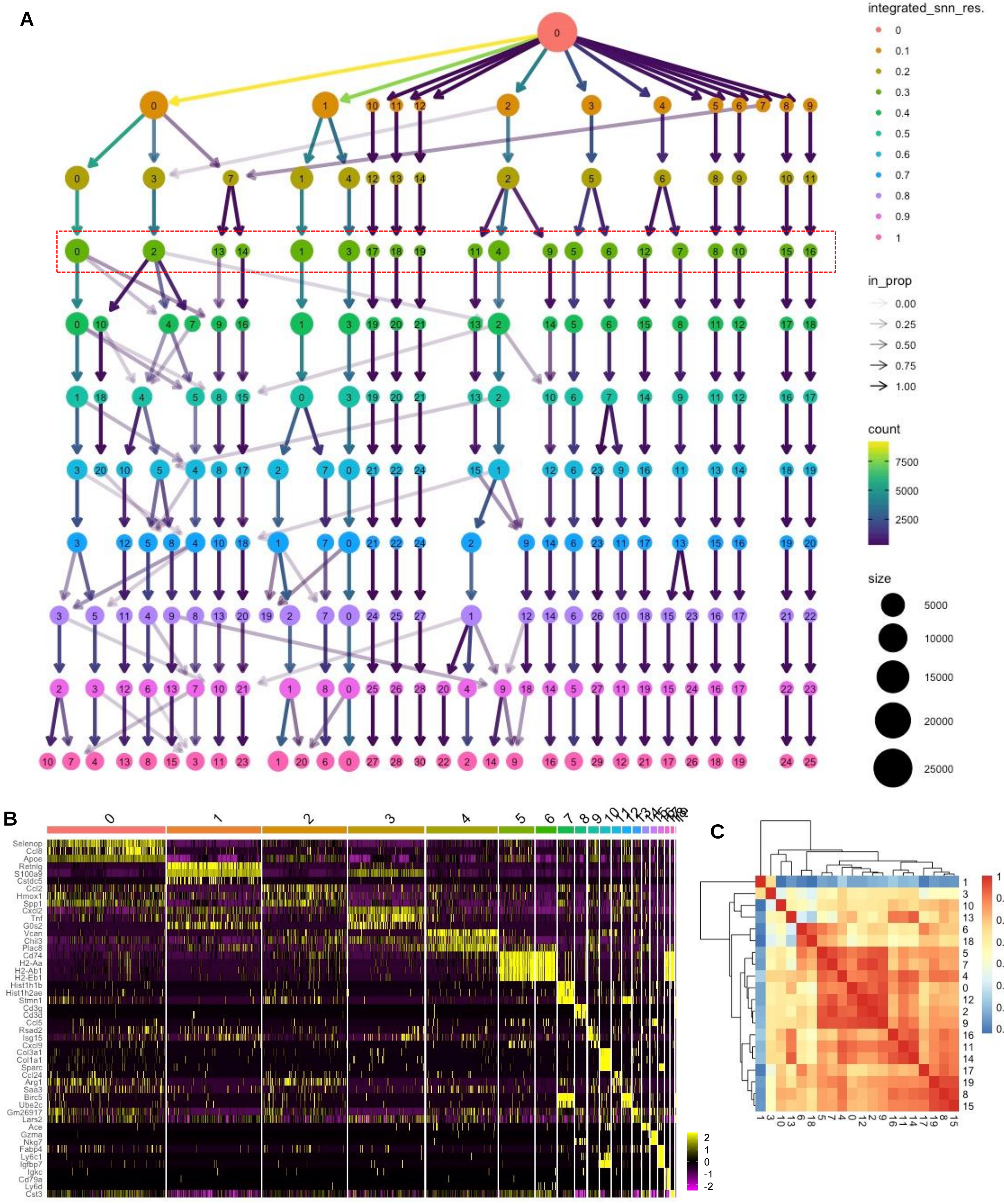
A
integrated_snn_res.
0
0.1
0.2
0.3
0.4
0.5
0.6
0.7
0.8
0.9
1
in_prop
0.00
0.25
0.50
0.75
1.00
count
7500
5000
2500
size
5000
10000
15000
20000
25000
B
Selenop
Ccl8
Apoe
Retnlg
S100a9
Cstdc5
Ccl2
Hmox1
Spp1
Cxcl2
Tnf
G0s2
Vcan
Chil3
Plac8
Cd74
H2-Aa
H2-Ab1
H2-Eb1
Hist1h1b
Hist1h2ae
Stmn1
Cd3g
Cd3d
Ccl5
Rsad2
Isg15
Cxcl9
Col3a1
Col1a1
Sparc
Ccl24
Arg1
Saa3
Birc5
Ube2c
Gm26917
Lars2
Ace
Gzma
Nkg7
Fabp4
Ly6c1
Igfbp7
Igkc
Cd79a
Ly6d
Cst3
2
1
0
-1
-2
C
1
0.9
0.8
0.7
0.6
0.5
0.4
0.3

Figure S2 (continued)

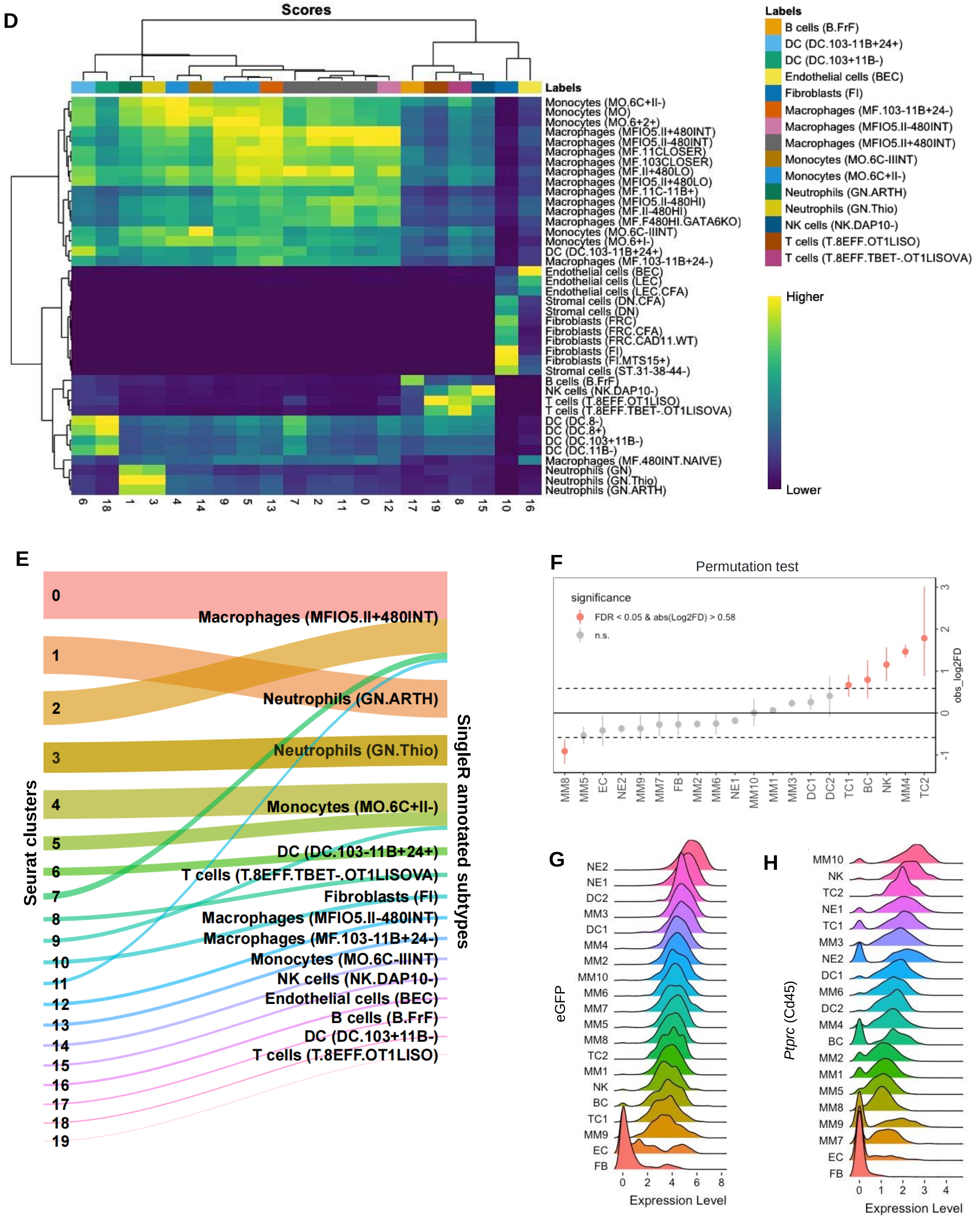

Figure S2. Cell clustering and phenotype annotation. (A) Clustree analysis of results from the Seurat clustering with resolution parameters ranging from 0.0 to 1.0. The circle color (coded by clustering resolution), intensity of in-proportion edges (indicating cell proportion derived from lower resolution), color of line edges (indicating cell numbers derived from lower resolution), and circle size (indicating the total cell number) are shown on the right. The red box indicates one stabile region at resolution-0.3 shown with few numbers of low in-proportion edges (0-0.25). (B) Heatmap showing the top 3 most differentially expressed genes in each cell cluster irrespective of graft sources. Average expression scale is shown on the right. (C) Heatmap representing Pearson correlations between the identified 20 cell clusters. Pearson correlation coefficient is shown on the right. (D) Heatmap showing the SingleR scores for all cells across all reference labels in the ImmGen dataset. The score scale is shown on the right and the cell labels are shown at the bottom. (E) Sankey plot colored by the Seurant clusters corresponding to the cell-type labels assigned from the SingleR annotations. Thicker line represents more cell numbers. (F) Comparison of cell proportions between syngrafts and allografts. The red dot indicates a significant change (FDR < 0.05 and fold change > 1.5) and the grey dot indicates no significant change (ns) (scProportionTest). (G-H) Ridge plots of *eGFP* and *Ptprc* (encoding Cd45) gene expressions in each cell type. Color is coded according to the cell type that is ordered by the gene expression level.

Figure S3

A

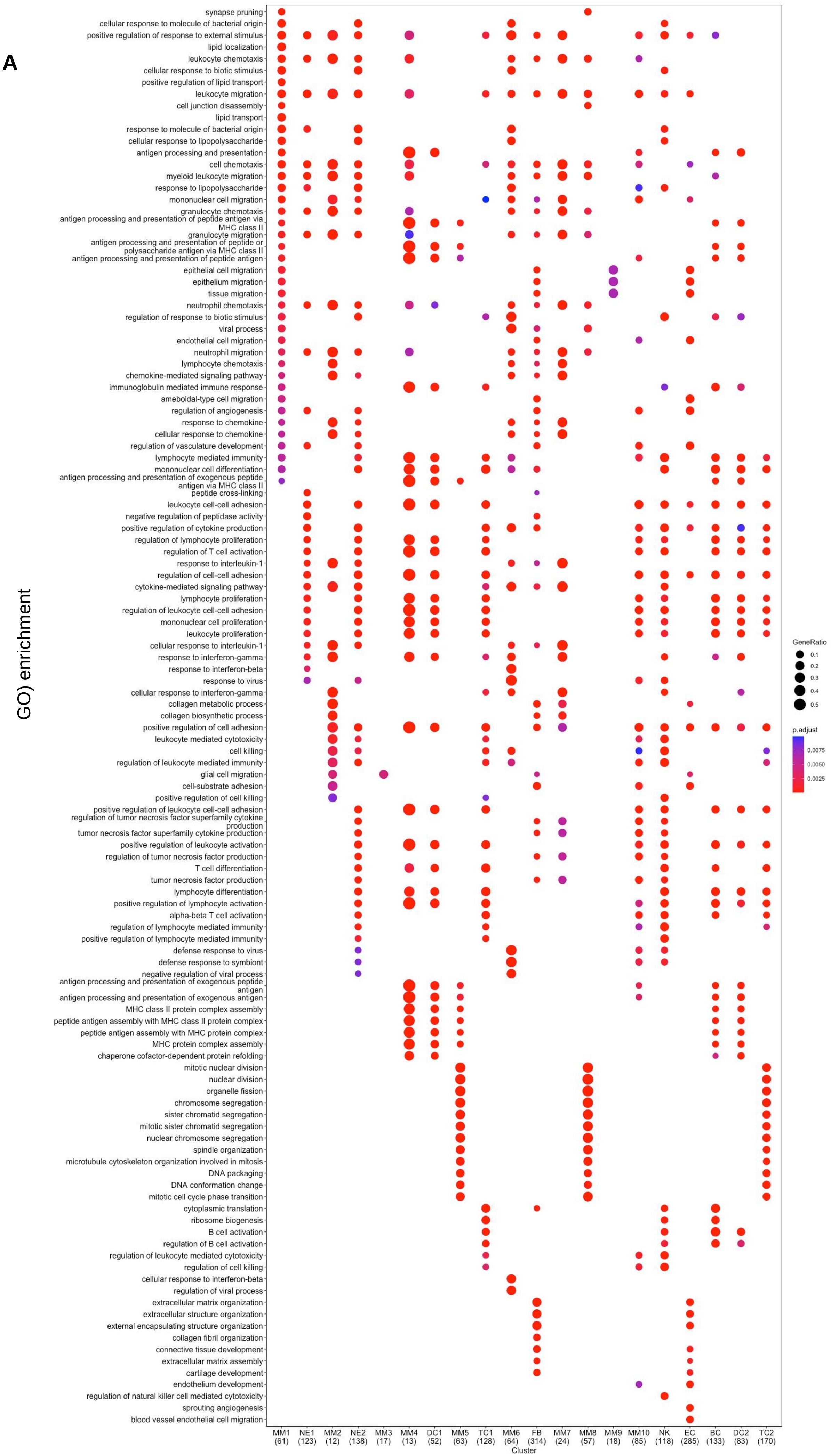

GO) enrichment
synapse pruning
cellular response to molecule of bacterial origin
positive regulation of response to external stimulus
lipid localization
leukocyte chemotaxis
cellular response to biotic stimulus
positive regulation of lipid transport
leukocyte migration
cell junction disassembly
lipid transport
response to molecule of bacterial origin
cellular response to lipopolysaccharide
antigen processing and presentation
cell chemotaxis
myeloid leukocyte migration
response to lipopolysaccharide
mononuclear cell migration
granulocyte chemotaxis
antigen processing and presentation of peptide antigen via MHC class II
granulocyte migration
antigen processing and presentation of peptide or polysaccharide antigen via MHC class II
antigen processing and presentation of peptide antigen
epithelial cell migration
epithelium migration
tissue migration
neutrophil chemotaxis
regulation of response to biotic stimulus
viral process
endothelial cell migration
neutrophil migration
lymphocyte chemotaxis
chemokine-mediated signaling pathway
immunoglobulin mediated immune response
ameboidal-type cell migration
regulation of angiogenesis
response to chemokine
cellular response to chemokine
regulation of vasculature development
lymphocyte mediated immunity
mononuclear cell differentiation
antigen processing and presentation of exogenous peptide antigen via MHC class II
peptide cross-linking
leukocyte cell-cell adhesion
negative regulation of peptidase activity
positive regulation of cytokine production
regulation of lymphocyte proliferation
regulation of T cell activation
response to interleukin-1
regulation of cell-cell adhesion
cytokine-mediated signaling pathway
lymphocyte proliferation
regulation of leukocyte cell-cell adhesion
mononuclear cell proliferation
leukocyte proliferation
cellular response to interleukin-1
response to interferon-gamma
response to interferon-beta
response to virus
cellular response to interferon-gamma
collagen metabolic process
collagen biosynthetic process
positive regulation of cell adhesion
leukocyte mediated cytotoxicity
cell killing
regulation of leukocyte mediated immunity
glial cell migration
cell-substrate adhesion
positive regulation of cell killing
positive regulation of leukocyte cell-cell adhesion
regulation of tumor necrosis factor superfamily cytokine production
tumor necrosis factor superfamily cytokine production
positive regulation of leukocyte activation
regulation of tumor necrosis factor production
T cell differentiation
tumor necrosis factor production
lymphocyte differentiation
positive regulation of lymphocyte activation
alpha-beta T cell activation
regulation of lymphocyte mediated immunity
positive regulation of lymphocyte mediated immunity
defense response to virus
defense response to symbiont
negative regulation of viral process
antigen processing and presentation of exogenous peptide antigen
antigen processing and presentation of exogenous antigen
MHC class II protein complex assembly
peptide antigen assembly with MHC class II protein complex
peptide antigen assembly with MHC protein complex
MHC protein complex assembly
chaperone cofactor-dependent protein refolding
mitotic nuclear division
nuclear division
organelle fission
chromosome segregation
sister chromatid segregation
mitotic sister chromatid segregation
nuclear chromosome segregation
spindle organization
microtubule cytoskeleton organization involved in mitosis
DNA packaging
DNA conformation change
mitotic cell cycle phase transition
cytoplasmic translation
ribosome biogenesis
B cell activation
regulation of B cell activation
regulation of leukocyte mediated cytotoxicity
regulation of cell killing
cellular response to interferon-beta
regulation of viral process
extracellular matrix organization
extracellular structure organization
external encapsulating structure organization
collagen fibril organization
connective tissue development
extracellular matrix assembly
cartilage development
endothelium development
regulation of natural killer cell mediated cytotoxicity
sprouting angiogenesis
blood vessel endothelial cell migration
MM1 (61)
NE1 (123)
MM2 (12)
NE2 (138)
MM3 (17)
MM4 (13)
DC1 (52)
MM5 (63)
TC1 (128)
MM6 (64)
FB (314)
MM7 (24)
MM8 (57)
MM9 (18)
MM10 (85)
NK (118)
EC (285)
BC (133)
DC2 (83)
TC2 (170)
Cluster
GeneRatio
0.1
0.2
0.3
0.4
0.5
p.adjust
0.0075
0.0050
0.0025

Figure S3 (continued)

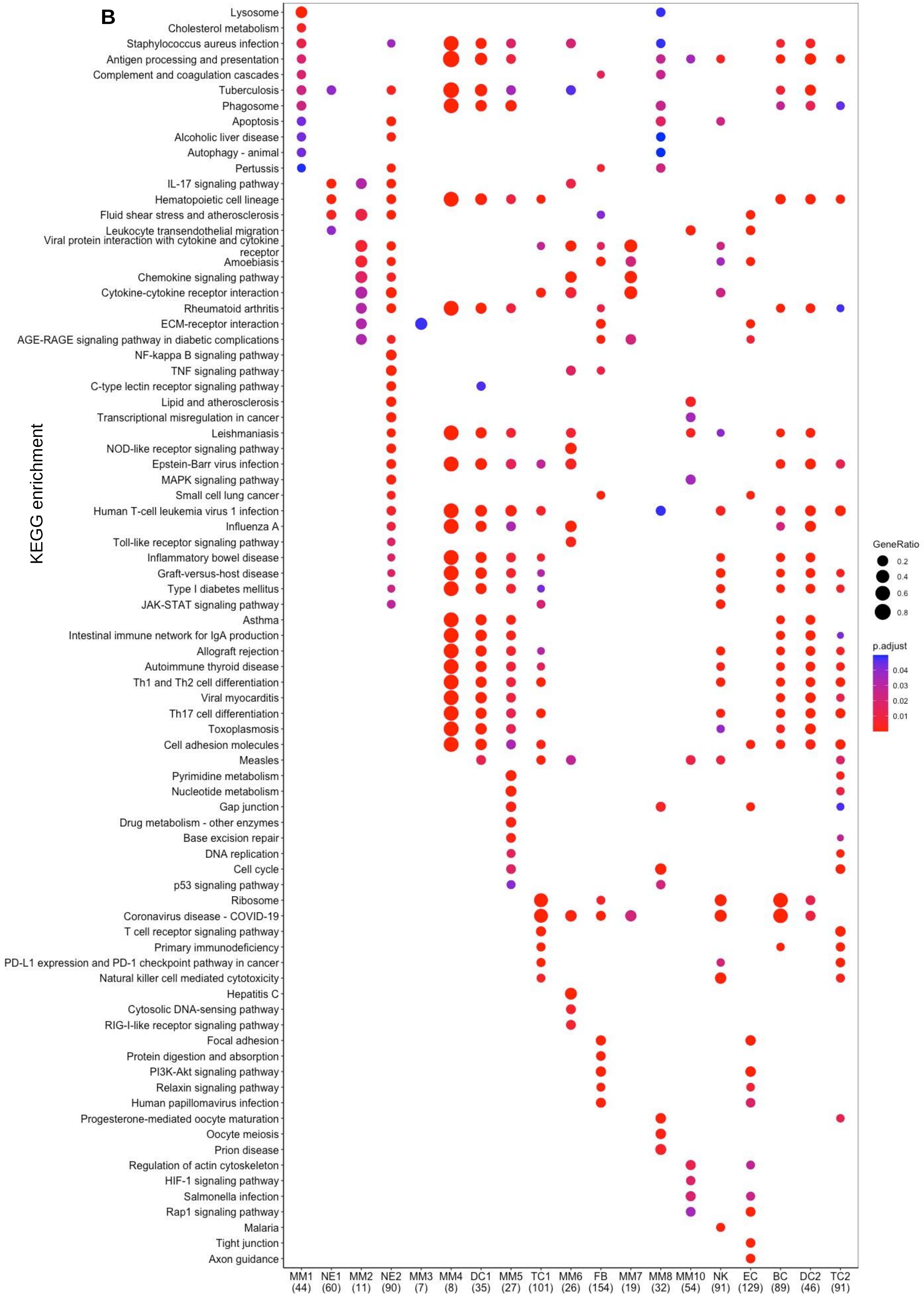

Figure S3. Functional annotation of infiltrating cells. (A-B) Gene ontology (GO) analysis and KEGG pathway analysis of the Seurat-identified marker genes of each cell type. The circle size indicates the ratio of gene activation in each GO term or signaling pathway. The scale of adjusted *p* value is shown on the right (using clusterProfiler package).

Figure S4

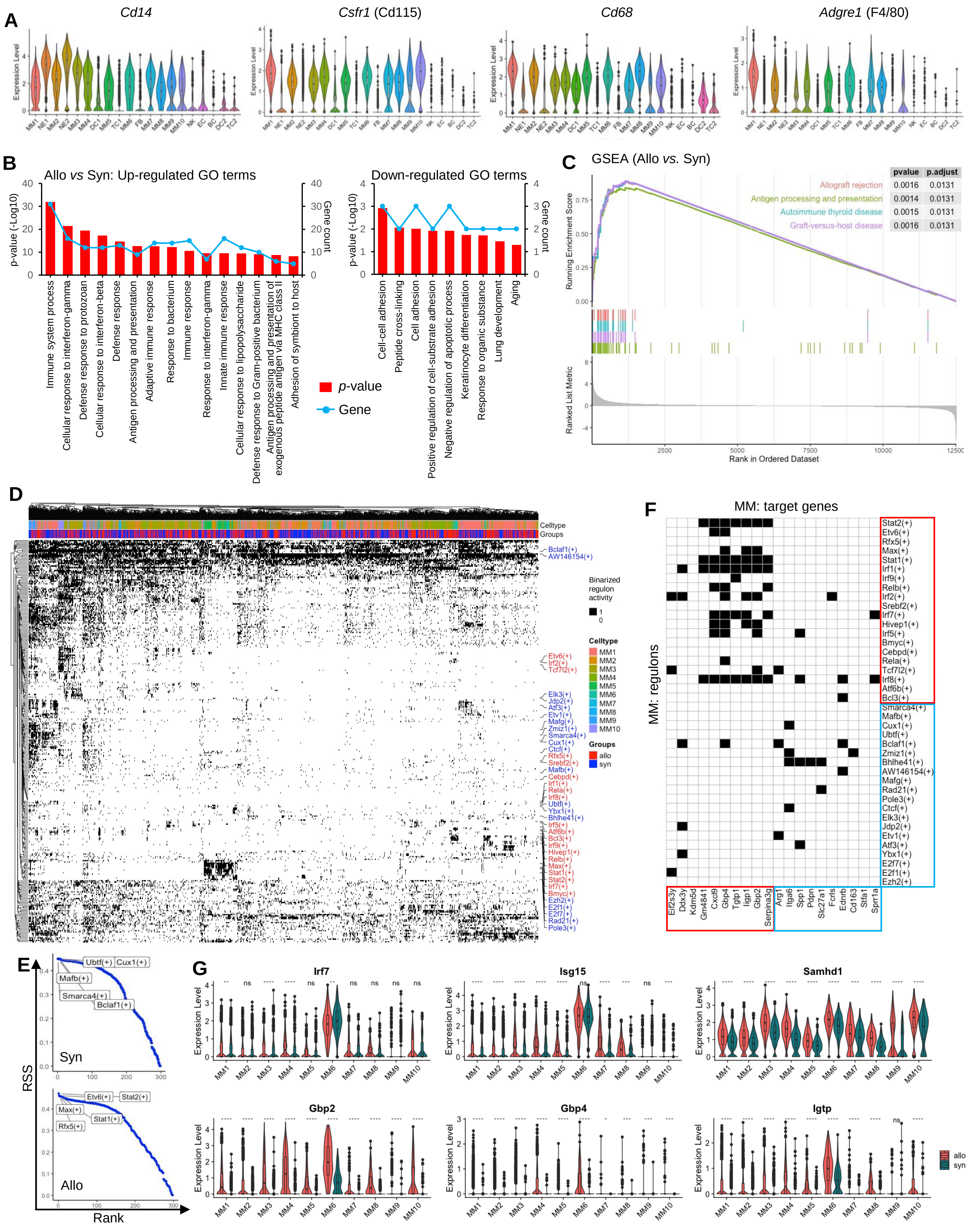
A
Cd14
Csfr1 (Cd115)
Cd68
Adgre1 (F4/80)
Expression Level
B
Allo vs Syn: Up-regulated GO terms
Down-regulated GO terms
p-value (-Log10)
Gene count
Immune system process
Cellular response to interferon-gamma
Defense response to protozoan
Cellular response to interferon-beta
Defense response
Antigen processing and presentation
Adaptive immune response
Response to bacterium
Immune response
Response to interferon-gamma
Innate immune response
Cellular response to lipopolysaccharide
Defense response to Gram-positive bacterium
Antigen processing and presentation of exogenous peptide antigen via MHC class II
Adhesion of symbiont to host
Cell-cell adhesion
Peptide cross-linking
Cell adhesion
Positive regulation of cell-substrate adhesion
Negative regulation of apoptotic process
Keratinocyte differentiation
Response to organic substance
Lung development
Aging
p-value
Gene
C
GSEA (Allo vs. Syn)
pvalue p.adjust
Allograft rejection 0.0016 0.0131
Antigen processing and presentation 0.0014 0.0131
Autoimmune thyroid disease 0.0015 0.0131
Graft-versus-host disease 0.0016 0.0131
Running Enrichment Score
Ranked List Metric
Rank in Ordered Dataset
D
Celltype
Groups
Binarized regulon activity
Celltype
MM1 MM2 MM3 MM4 MM5 MM6 MM7 MM8 MM9 MM10
Groups
allo
syn
E
RSS
Syn
Allo
Rank
F
MM: target genes
MM: regulons
G
Irf7
Isg15
Samhd1
Gbp2
Gbp4
Igtp
allo
syn

Figure S4. Gene expression profiles of monocytes and macrophages. (A) Violin plots showing normalized expression levels of the macrophage markers such as *Cd14*, *Csfr1*, *Cd68*, and *Adgre1* genes in all cell subsets. (B) GO terms related to upregulated or downregulated genes in pseudobulk samples of monocytes and macrophages in allografts as compared with syngrafts. The gene count and adjusted *p* value (from the DAVID functional annotation) are shown in the chart. (C) Gene set enrichment analysis (GSEA) plot of the top four KEGG pathways activated in pseudobulk samples of monocytes and macrophages in allografts as compared with syngrafts. The bars underneath the lines of running enrichment score show where the members of the gene set of a pathway appear in the ranked list of genes. The ranking metric indicates the fold change of a ranked gene. (D) Heatmap showing a binarized activity matrix of transcription factor (TF)-gene regulons in each cell of MM subsets by the SCENIC analysis. The color depiction of cell type, sample source, and regulon activity is shown on the right. (E) The top five regulons in monocytes and macrophages from syngrafts or allografts. Rank for regulon is based on its specificity score (RSS). (F) A heatmap showing the potential transcription factors/regulators (rows) for given genes (columns) in monocytes and macrophages. Red box highlights allograft-specific genes and blue box highlights syngraft-specific genes. Back color indicates TF motif-targeting and white color indicates non-targeting. (G) Violin plots showing normalized expression levels of the interferon (IFN-I or IFN-II)-related genes in each monocyte and macrophage subset from syngrafts or allografts. ^{ns} not significant, ** $p<0.01$, *** $p<0.001$, **** $p<0.0001$ (Student's t-test).

Figure S5

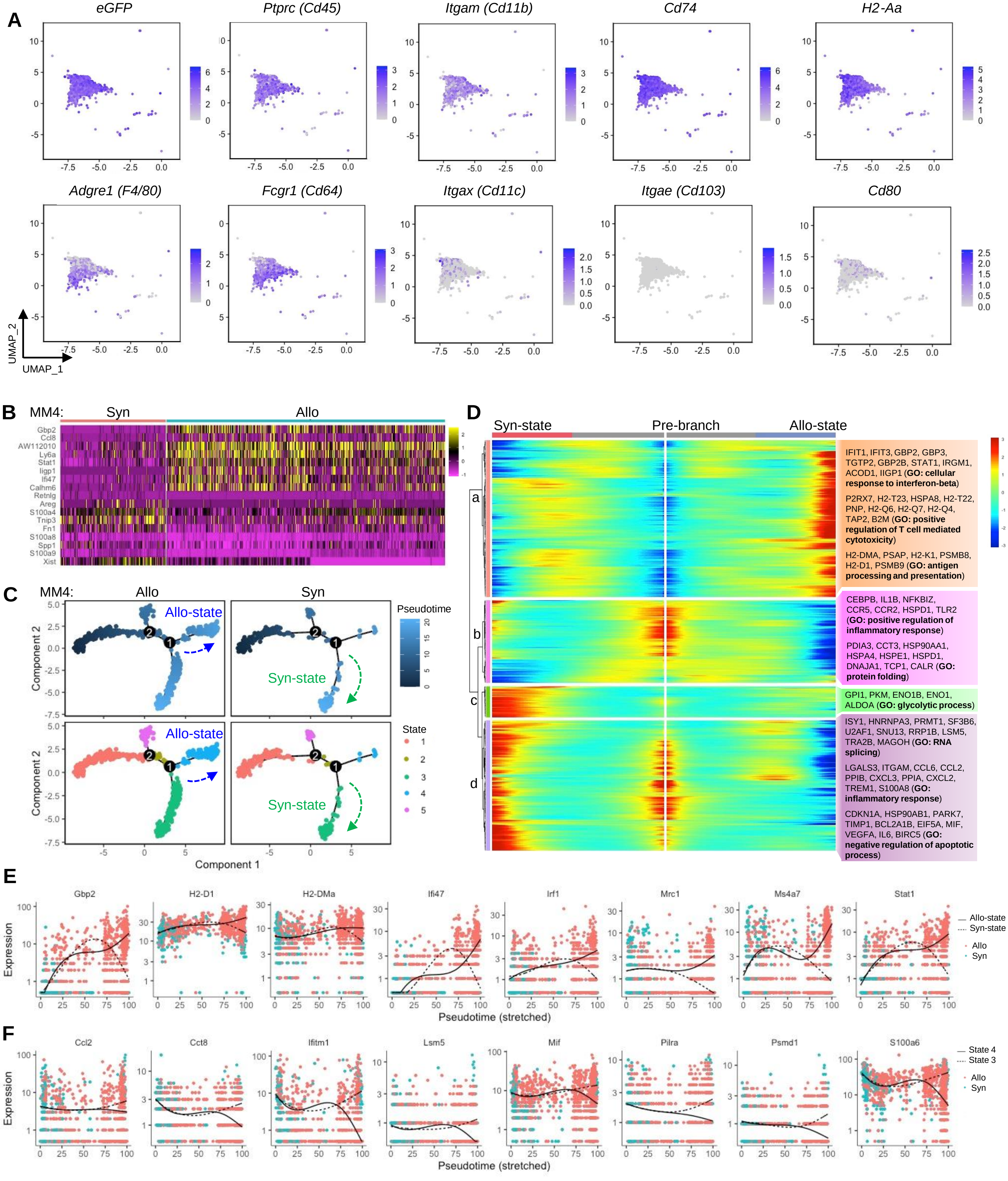
A
eGFP
Ptprc (Cd45)
Itgam (Cd11b)
Cd74
H2-Aa
Adgre1 (F4/80)
Fcgr1 (Cd64)
Itgax (Cd11c)
Itgae (Cd103)
Cd80
UMAP_2
UMAP_1
B
MM4:
Syn
Allo
Gbp2
Ccl8
AW112010
Ly6a
Stat1
Iigp1
Ifi47
Calhm6
Retnlg
Areg
S100a4
Tnip3
Fn1
S100a8
Spp1
S100a9
Xist
C
MM4:
Allo
Syn
Allo-state
Syn-state
Pseudotime
State
Component 2
Component 1
D
Syn-state
Pre-branch
Allo-state
a
b
c
d
IFIT1, IFIT3, GBP2, GBP3, TGTP2, GBP2B, STAT1, IRGM1, ACOD1, IIGP1 (GO: cellular response to interferon-beta)
P2RX7, H2-T23, HSPA8, H2-T22, PNP, H2-Q6, H2-Q7, H2-Q4, TAP2, B2M (GO: positive regulation of T cell mediated cytotoxicity)
H2-DMA, PSAP, H2-K1, PSMB8, H2-D1, PSMB9 (GO: antigen processing and presentation)
CEBPB, IL1B, NFKBIZ, CCR5, CCR2, HSPD1, TLR2 (GO: positive regulation of inflammatory response)
PDIA3, CCT3, HSP90AA1, HSPA4, HSPE1, HSPD1, DNAJA1, TCP1, CALR (GO: protein folding)
GPI1, PKM, ENO1B, ENO1, ALDOA (GO: glycolytic process)
ISY1, HNRNPA3, PRMT1, SF3B6, U2AF1, SNU13, RRP1B, LSM5, TRA2B, MAGOH (GO: RNA splicing)
LGALS3, ITGAM, CCL6, CCL2, PPIB, CXCL3, PPIA, CXCL2, TREM1, S100A8 (GO: inflammatory response)
CDKN1A, HSP90AB1, PARK7, TIMP1, BCL2A1B, EIF5A, MIF, VEGFA, IL6, BIRC5 (GO: negative regulation of apoptotic process)
E
Gbp2
H2-D1
H2-DMa
Ifi47
Irf1
Mrc1
Ms4a7
Stat1
Expression
Pseudotime (stretched)
Allo-state
Syn-state
Allo
Syn
F
Ccl2
Cct8
Ifitm1
Lsm5
Mif
Pilra
Psmd1
S100a6
Expression
Pseudotime (stretched)
State 4
State 3
Allo
Syn

Figure S5. Difference of the MM4 subset between syngrafts and allografts. (A) UMAP plots of various gene expressions (APC markers (e.g. *Cd74*, H2-Aa), macrophage genes (e.g. *F4/80*, *Cd64*). DC markers (e.g. *Cd11c*, *Cd103*)) in the MM4 subset (defined as monocyte-derived cells). (B) Heatmap showing the differential gene expression of MM4 between syngrafts and allografts. IFN-γ-related genes such as *Stat1*, *Ifi47*, and *Gbp2* are listed. Average expression scale is shown on the right. (C) Trajectory plots (upper) showing pseudotime distribution of MM4 in different graft types. Time scale is shown on the right. Pseudotime-0 indicates starting cells. Trajectory plots (lower) showing potential paths of MM4 activation in different graft types, colored according to the cell state. (D) Heatmap showing graft type-dependent gene expression patterns through comparing the Syn-state and Allo-state split from the branch point-1 of MM4 pseudo-trajectory. And gene ontology (GO) terms related to the gene profiles are shown on the right. (E-F) Scatter plots of the expression levels of representative genes upregulated or downregulated along the MM4 pseudo-trajectory. Color is coded according to the graft type. Solid line indicates Allo-state. Dotted line indicates Syn-state.

Figure S6

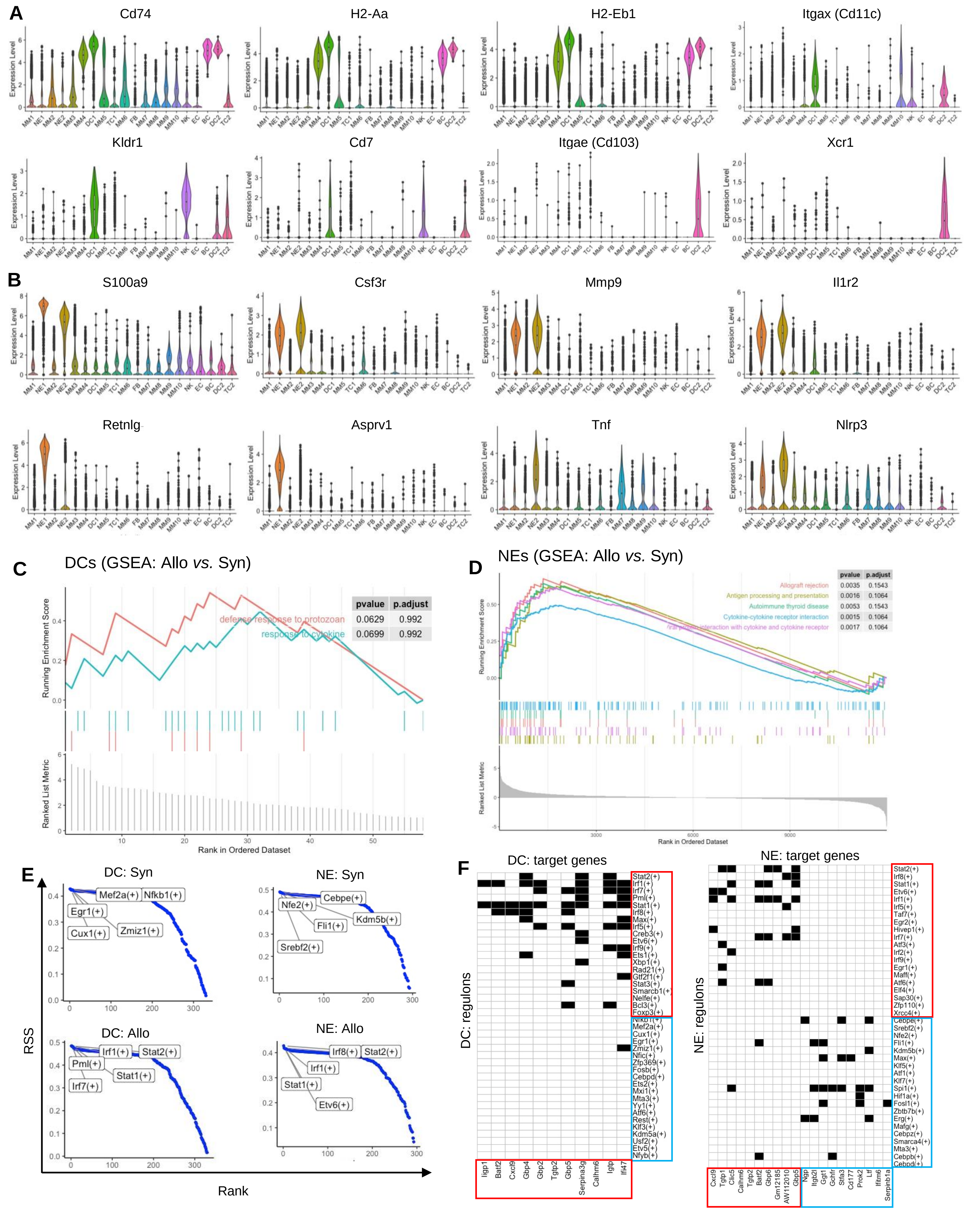

A
Cd74
H2-Aa
H2-Eb1
Itgax (Cd11c)
Kldr1
Cd7
Itgae (Cd103)
Xcr1
B
S100a9
Csf3r
Mmp9
Il1r2
Retnlg
Asprv1
Tnf
Nlrp3
C
DCs (GSEA: Allo vs. Syn)
defense response to protozoan
response to cytokine
pvalue
p.adjust
0.0629
0.992
0.0699
0.992
Running Enrichment Score
Ranked List Metric
Rank in Ordered Dataset
D
NEs (GSEA: Allo vs. Syn)
Allograft rejection
Antigen processing and presentation
Autoimmune thyroid disease
Cytokine-cytokine receptor interaction
E
DC: Syn
NE: Syn
DC: Allo
NE: Allo
RSS
Rank
F
DC: target genes
NE: target genes
DC: regulons
NE: regulons

Figure S6. Gene expression profiles of neutrophils and dendritic cells. (A-B) Violin plots showing normalized expression levels of the dendritic cell (A) or neutrophil (B) markers in all cell subsets. (C) GSEA plot of the top two KEGG pathways activated in pseudobulk samples of dendritic cells in allografts as compared with syngrafts. The bars underneath the lines of running enrichment score show where the members of the gene set of a pathway appear in the ranked list of genes. The ranking metric indicates the fold change of a ranked gene. (D) GSEA plot of the top five KEGG pathways activated in pseudobulk samples of neutrophils in allografts as compared with syngrafts. The bars underneath the lines of running enrichment score show where the members of the gene set of a pathway appear in the ranked list of genes. The ranking metric indicates the fold change of a ranked gene. (E) The top five regulons in dendritic cells (left panels) or neutrophils (right panels) from syngrafts or allografts. Rank for regulon is based on its specificity score (RSS). (F) Heatmap showing the potential transcription regulators (rows) for given genes (columns) in dendritic cells (left panel) or neutrophils (right panel). Back color indicates TF motif targeting and while color indicates non-targeting. Red box highlights allograft-specific genes and blue box highlights syngraft-specific genes.

Figure S7

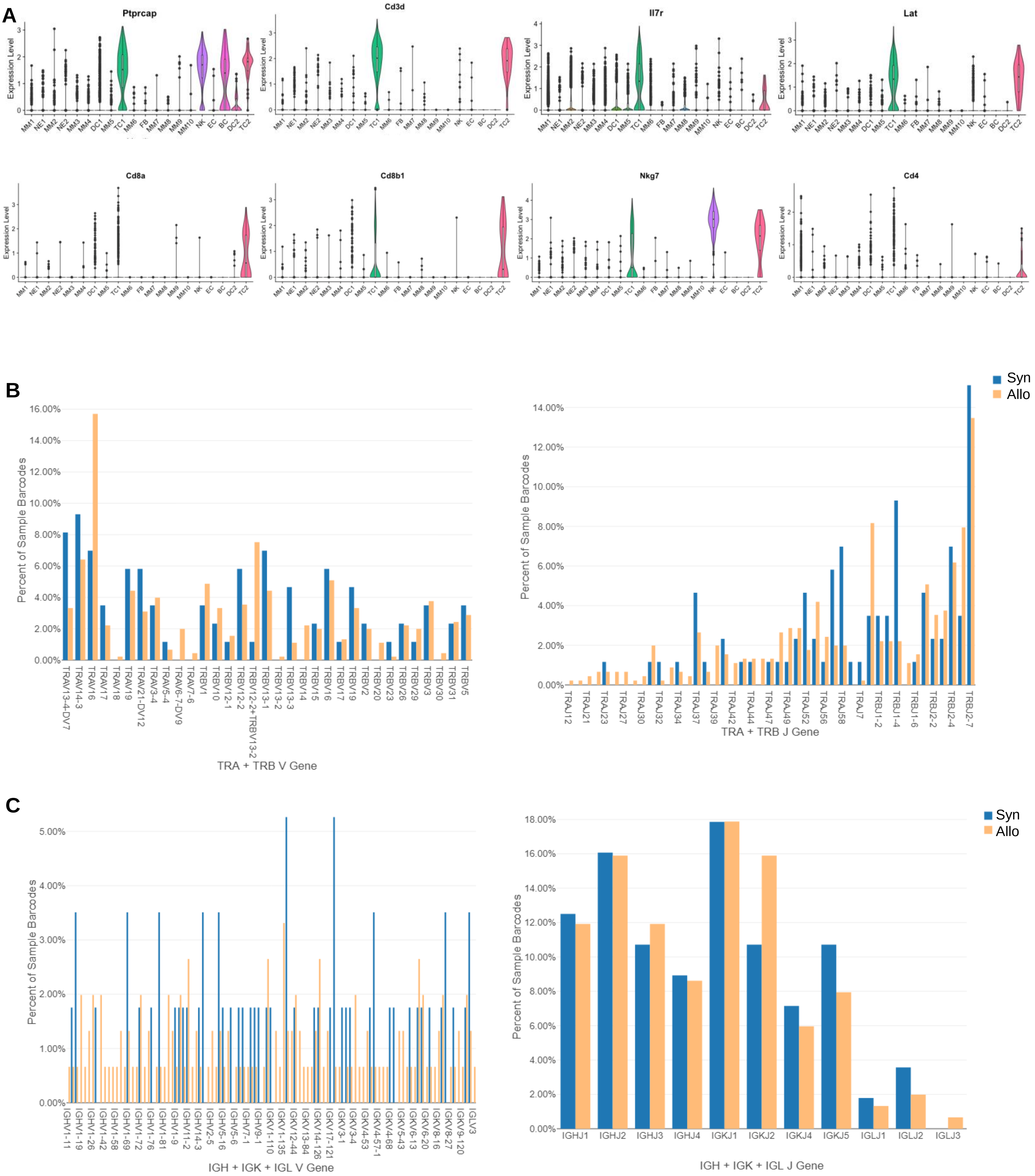
A
Ptprcap
Cd3d
Il7r
Lat
Cd8a
Cd8b1
Nkg7
Cd4
Expression Level
MM1 NE1 MM2 NE2 MM3 MM4 DC1 MM5 TC1 MM6 FB MM7 MM8 MM9 MM10 NK EC BC DC2 TC2
B
Syn
Allo
Percent of Sample Barcodes
TRA + TRB V Gene
TRAV13-4-DV7 TRAV14-3 TRAV16 TRAV17 TRAV18 TRAV19 TRAV21-DV12 TRAV3-4 TRAV5-4 TRAV6-7-DV9 TRAV7-6 TRBV1 TRBV10 TRBV12-1 TRBV12-2 TRBV12-2+TRBV13-2 TRBV13-1 TRBV13-2 TRBV13-3 TRBV14 TRBV15 TRBV16 TRBV17 TRBV19 TRBV2 TRBV20 TRBV23 TRBV26 TRBV29 TRBV3 TRBV30 TRBV31 TRBV5
TRA + TRB J Gene
TRAJ12 TRAJ21 TRAJ23 TRAJ27 TRAJ30 TRAJ32 TRAJ34 TRAJ37 TRAJ39 TRAJ42 TRAJ44 TRAJ47 TRAJ49 TRAJ52 TRAJ56 TRAJ58 TRAJ7 TRBJ1-2 TRBJ1-4 TRBJ1-6 TRBJ2-2 TRBJ2-4 TRBJ2-7
C
Syn
Allo
Percent of Sample Barcodes
IGH + IGK + IGL V Gene
IGHV1-11 IGHV1-19 IGHV1-26 IGHV1-42 IGHV1-58 IGHV1-69 IGHV1-72 IGHV1-76 IGHV1-81 IGHV1-9 IGHV11-2 IGHV14-3 IGHV2-5 IGHV5-16 IGHV5-6 IGHV7-1 IGHV9-1 IGKV1-110 IGKV1-135 IGKV12-44 IGKV13-84 IGKV14-126 IGKV17-121 IGKV3-1 IGKV3-4 IGKV4-53 IGKV4-57-1 IGKV4-68 IGKV5-43 IGKV6-13 IGKV6-20 IGKV8-16 IGKV8-27 IGKV9-120 IGLV3
IGH + IGK + IGL J Gene
IGHJ1 IGHJ2 IGHJ3 IGHJ4 IGKJ1 IGKJ2 IGKJ4 IGKJ5 IGLJ1 IGLJ2 IGLJ3

Figure S7. T and B lymphocytes and natural killer cells. (A) Violin plot showing normalized expression levels of the lymphocyte markers in all cell subsets. (B) Bar charts showing the percentage of cell barcodes in various T cell clonotypes that are distinguished by different recombination of V (left panel) or J (right panel) genes in TRA (T cell receptor alpha locus) and TRB (T cell receptor beta locus). (C) Bar charts showing the percentage of cell barcodes in various B cell clonotypes that are distinguished by different recombination of V (left panel) or J (right panel) genes in immunoglobulin loci (IGH, IGK, and IGL).

Figure S8

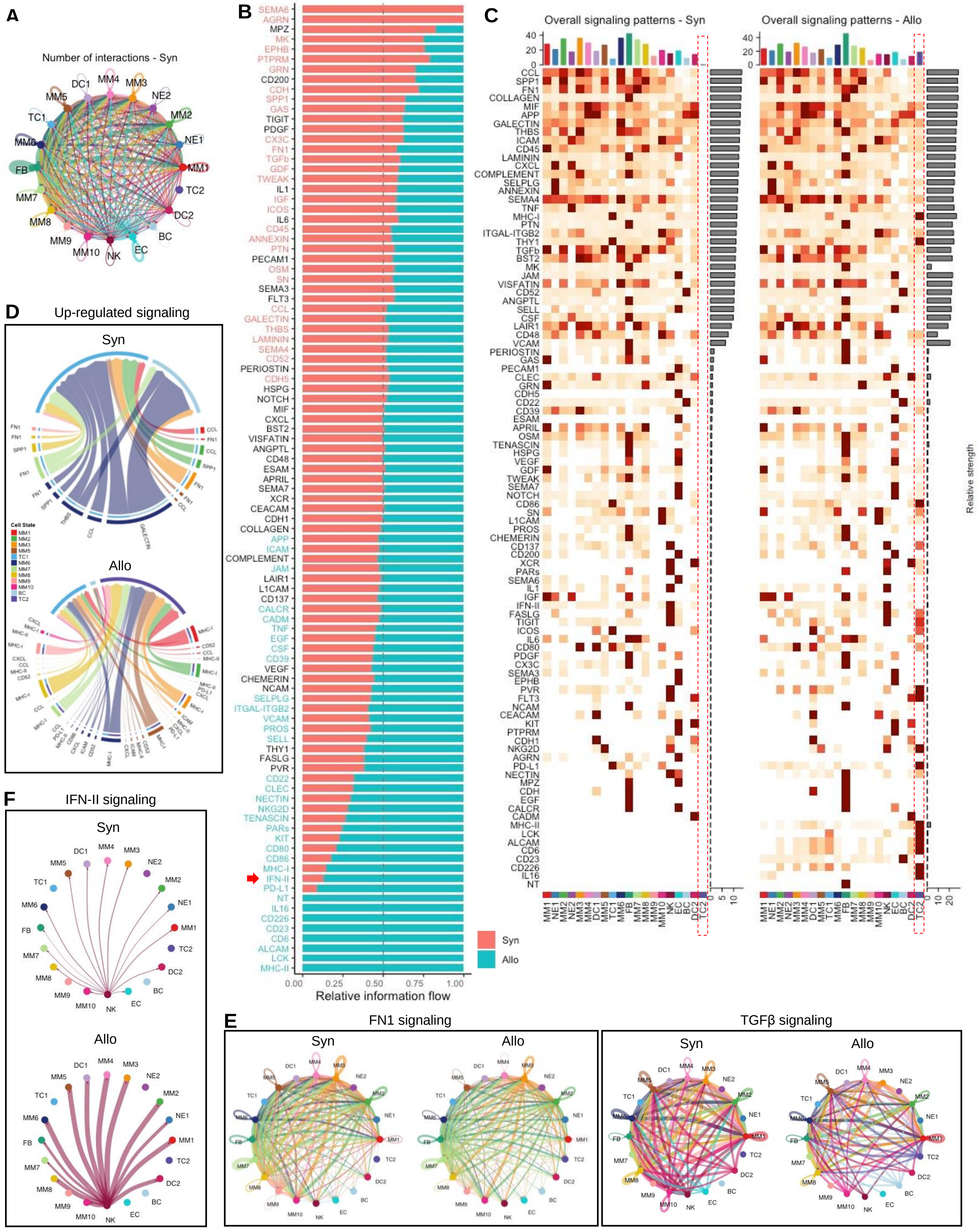
A
Number of interactions - Syn
B
Relative information flow
Syn
Allo
C
Overall signaling patterns - Syn
Overall signaling patterns - Allo
Relative strength
D
Up-regulated signaling
Syn
Allo
Cell State
E
FN1 signaling
TGFβ signaling
Syn
Allo
F
IFN-II signaling
Syn
Allo

Figure S8. Profiles of cell-cell communication network between infiltrating cells. (A) circle plot showing the number of cell-cell interactions (including autocrine and paracrine) in synografts. Circle color is coded according to the cell type and line color indicates the cell source as the sender. Connected line indicates autocrine or paracrine signaling. Thicker line represents more number of interactions. (B) Bar graph showing all significant signaling pathways ranked based on their differences in overall information flow within the inferred networks between allografts and syngrafts. The top signaling pathways colored red are enriched in syngrafts, the bottom ones colored green are enriched in allografts, and the middle ones colored black are equally enriched in both two graft types. (C) Heatmaps showing overall (including incoming and outgoing) signaling pathways identified from syngraft (left panel) and allograft (right panel) datasets. The color indicates the signaling relative strength as shown on the right. (D) Chord diagrams showing upregulated signaling of ligand-receptor pairs sent from monocytes and macrophages (MM1-10) to T cells and B cells in syngrafts or allografts. Color is coded according to the cell type. Edge colors are consistent with the sources as sender. The arrowhead indicates the targets (the inner thinner bars) that receive a signal from the corresponding outer bars. The inner bar size is proportional to the signal strength received by the targets. (E) Circle plots showed the intercellular communication network of tissue repair-related signaling pathways (FN1 and TGFβ) in syngrafts or allografts. Circle sizes are proportional to the number of cells in each cell group and edge width represents the communication probability. (F) Circle plots showed the intercellular communication network of IFN-II signaling pathway in syngrafts or allografts. Circle sizes are proportional to the number of cells in each cell group and edge width represents the communication probability.

Figure S9

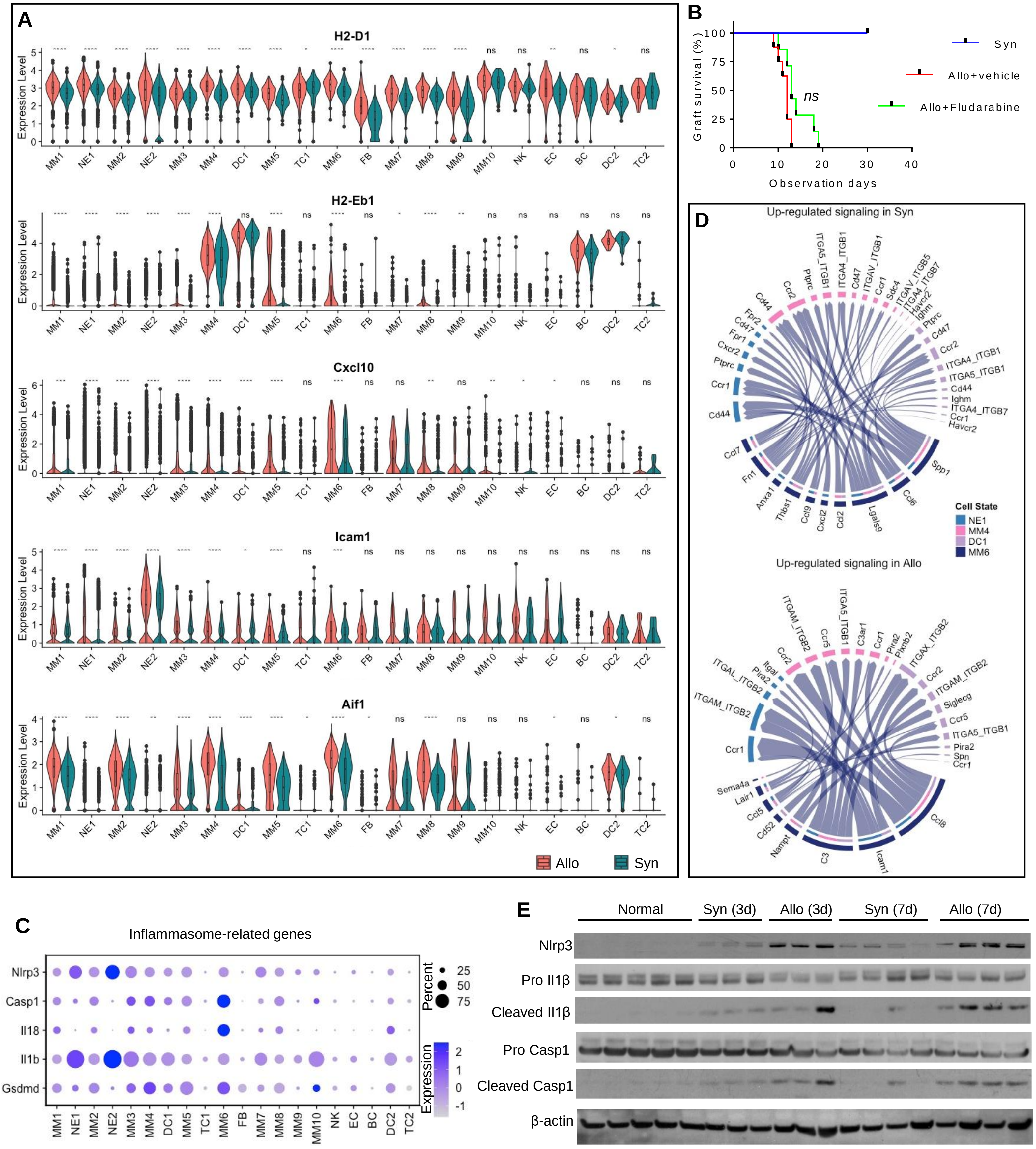

A
H2-D1
H2-Eb1
Cxcl10
Icam1
Aif1
Expression Level
MM1 NE1 MM2 NE2 MM3 MM4 DC1 MM5 TC1 MM6 FB MM7 MM8 MM9 MM10 NK EC BC DC2 TC2
ns
Allo
Syn
B
Graft survival (%)
Observation days
ns
Syn
Allo+vehicle
Allo+Fludarabine
C
Inflammasome-related genes
Nlrp3
Casp1
Il18
Il1b
Gsdmd
Percent
Expression
D
Up-regulated signaling in Syn
Up-regulated signaling in Allo
Cell State
NE1
MM4
DC1
MM6
E
Normal
Syn (3d)
Allo (3d)
Syn (7d)
Allo (7d)
Nlrp3
Pro Il1β
Cleaved Il1β
Pro Casp1
Cleaved Casp1
β-actin

Figure S9. Involvement of inflammasome in rejection. (A) Violin plot showing the differential expression level of interferon-γ-related downstream genes (*H2-K1* (MHC-I), *H2-Eb1* (MHC-II), *Cxcl10* (chemotaxis), *Icam1* (adhesion), and *Aif1* (inflammation)) in the infiltrating cells of allografts as compared with syngrafts. ^{ns} not significant, * $p<0.05$, ** $p<0.01$, *** $p<0.001$, **** $p<0.0001$ (Student's t-test). (B) Survival curves of syngrafts and allografts with or without treatment (median survival time: 13 days *vs*. 12 days, $p= 0.0587$). The complete cessation of a heartbeat is the endpoint. n=7~8 per group. ^{ns} *vs* allograft+vehicle, not significant (Gehan-Breslow-Wilcoxon test). (C) A dot plot showing the expressions of inflammasome-related genes in all cell clusters. The scale of dot size representing cell percentage and dot color representing the average expression is shown on the right. (D) Chord diagrams showing upregulated signaling of ligand-receptor pairs sent from inflammatory monocytes (MM6) to representative antigen-processing cells (DC2 and MM4) and neutrophils (NE1) in syngrafts or allografts. Color is coded according to the cell type. Edge colors are consistent with the sources as sender. The arrowhead indicates the targets (the inner thinner bars) that receive a signal from the corresponding outer bars. The inner bar size is proportional to the signal strength received by the targets. (E) Western blotting of Nlrp3, Il1β, and caspase-1 expressions in normal, syngraft, or allograft hearts at 3 or 7 days post-transplant.
