# Supplementary material for: Single-cell analysis of graft-infiltrating host cells identifies caspase-1 as a potential therapeutic target for heart transplant rejection
Source: Front Immunol. 2023 Sep 13;14:1251028. doi: 10.3389/fimmu.2023.1251028 (PMC10535112; doi:10.3389/fimmu.2023.1251028)
Supplement: Supplementary file 2 [file DataSheet_2.docx]

**Supplemental materials and methods:**

**scRNAseq data normalization and integration**

The sequencing data of 4 samples were preprocessed using the Cell Ranger Single-Cell Software v6.0 (10×genomics) according to the manufacturer’s instructions. Raw files generated by the sequencer were demultiplexed using *cellranger mkfastq* and FASTQ files were generated. For mapping reads from tracing experiments, the mm10 reference genome was customized with the addition of the eGFP sequence that was used to create the mouse line([1](#_ENREF_1)). The eGFP sequence was annotated in the GTF (gene transfer format) file and the reference mm10 genome was rebuilt using the *cellranger* *mkref* command. Then, the collected FASTQ files were analyzed using *cellranger count* for alignment, filtering, barcode counting, and UMI counting. Finally, the Cell Ranger outputs including feature-barcode matrices were used to determine cell clusters and perform gene expression analysis.

The secondary analyses were performed on the platform of R v4.1.2 or Python v3.9.7 and the default settings were applied in the programs, if not explicitly stated otherwise. Firstly, the 10× scRNAseq data were analyzed using Seurat v4.1.1 according to the published tutorial([2](#_ENREF_2)). After loading the cell matrix data, the Seurat object for each sample was created. Then, the data quality control was performed according to the standard pre-processing workflow. The low-quality cells that have unique feature counts less than 200 or over 6000 and >10% mitochondrial counts were filtered out. After data normalizing, scaling, and dimensional reduction for each library, DoubletFinder([3](#_ENREF_3)) was used to filter out the potential doublets and the singlets were used for the downstream analysis. Furthermore, all datasets were merged and the experiment design information such as the batch number and sample name was added to the metadata. The reciprocal principal component analysis (PCA) was utilized to identify anchors for dataset integration([4](#_ENREF_4)).

**Cell clustering**

The integrated dataset was analyzed by running PCA and UMAP in the standard workflow of Seurat for visualization and clustering. To fully uncover cell transcriptional state heterogeneity while avoiding “false heterogeneity” with poorly defined cell clusters, Clustree R Bioconductor package([5](#_ENREF_5)) together with the *FindClusters* function in Seurat was used to create a plot of a clustering tree showing the relationship between clusters at increasing resolutions (0.0-1.0) and validating the legitimacy of the clusters using classification. Based on these two algorithms, we used a value of 0.3 for the resolution and obtained 20 cell clusters. Furthermore, dimensional reduction (UMAP) plots were generated by using the *DimPlot* function in Seurat package and the cells can be labeled with different colors according to the needed metadata. To assess the similarity and compute the cross-correlation among cell clusters, Pearson correlation coefficient was calculated between the average expression of genes in each cell of each cluster using the *cor* function in R programming and visualized by the pheatmap package.

**Cell type annotation**

To unbiasedly classify the cell phenotypes, the SingleR pipeline which is an automatic annotation method([6](#_ENREF_6)) was used to annotate the Seurat-identified cell clusters. The Immunological Genome Project (ImmGen) database([7](#_ENREF_7)) was used as a reference dataset for mouse sample analysis in this study. The SingleR-calculated scores for all cells across all reference labels were shown in a heatmap using the *plotScoreHeatmap* function. Furthermore, the SingleR-annotated cell phenotype was confirmed manually according to the conserved marker genes that were identified using the *FindConservedMarkers* function in Seurat. The Seurat clusters that shared the same SingleR-annotation showed different marker profiles, therefore we named the cell subsets using the main cell type plus different numbers. A river flow plot was generated by using the *getSankey* function of the scmap R package([8](#_ENREF_8)) to show the relationship between the Seurat clusters and SingleR annotation.

**Differential cell proportion analysis**

The difference between the proportions of cells in clusters between syngrafts and allografts was analyzed using the scProportionTest R package (https://github.com/rpolicastro/scProportionTest/). A permutation test was used to calculate a p-value for each Seurat cluster, and a confidence interval for the magnitude difference (log2(fold changes)) was returned via bootstrapping. A point-range plot of the results then was created. Furthermore, the data frame of cluster cell numbers from the four graft samples was created and the cell proportion of each cell cluster was visualized by using the ggplot2 R package.

**Marker gene identification**

Markers specific to each cluster were identified using the *FindAllMarkers* function in Seurat with the following settings (only.pos=TRUE, logfc.threshold=1, and the rest was the default setting). The detailed marker gene list was available in Supplemental File 1. The cluster-specific markers were shown in a heatmap using the DoHeatmap function. Furthermore, the marker gene expressions in different subsets were visualized in dot plots using the *DotPlot* function, in ridge plots using the *RidgePlot* function, or in violin plots using the *VlnPlot* function. For comparing the differential expression in each cell cluster between syngrafts and allografts, Student’s t-test was performed in the plots using the *stat_compare_means* function from the ggpubr R package. All results of cell clusters and marker genes are available in the supplemental **File S1**.

**Gene signature scoring**

To enhance the cell resolution, different cell clusters were extracted from the integrated Seurat data by using the *subset* function for further analysis. The UCell R package was used to evaluate the gene signature enrichment of each cell type on basis of a list of reference gene sets. For monocytes and macrophages (MMs), the reference gene sets included M1 (Nos2, Il1a, Cd80, Cd86, Ccl19, Isg15, Irf7, Il12b, Il12a, Tnf, Il6), M2 (Mrc1, Arg1, Ly6c1, Ccl8, Il1r2, Maf, Chil3, Ido1, Retnla, Il10, Cd163), M_IFN-γ (Stat1, Socs1, Ido1, Nos2), and M_IL10 (Nfil3, Sbno2, Socs3, Il10, Il4ra). For dendritic cells (DCs), the reference gene sets included cDC1 (Xcr1, Itgae, Cd24a, Clec9a, Batf3, Irf8, Id2, Nfil3), cDC2 (Sirpa, Itgam, Irf4, Irf2), pDC (Siglech, Bst2, Cd209a, Ccr9, Ly6c1, Ifnb1), and moDC (Itgax, Adgre1, Fcgr1). For neutrophils (NEs), the reference gene sets included N1 (Ccl3, Ccl5, Il12a, Tnf, Il1b, Il6) and N2 (Mrc1, Arg1, Il10, Tgfb1, Chi3l1). For T cells, the reference gene sets included naive (Il7r, Sell, Il2rg, Ccr7) and effector-cytotoxicity (Gzmb, Gzma, Pdcd1). To assess the responses to interferons, the gene lists of IFN-I signaling (GO:0034340) and IFN-II signaling (GO:0034341) were downloaded from the MSigDB mouse database and the cell signatures were scored using the UCell analysis. The UCell scoring results were visualized in violin plots.

**Pseudobulk differential expression analysis**

Distinct from Seurat’s identification of marker genes at the individual cell level, it would be important to perform differential expression analysis between conditions (allografts vs syngrafts, in this study) within particular cell types. After confirming the cell identity, a pseudobulk approach was used to multiplex individual cells as one population/sample allowing for differential expression analysis between different conditions([9](#_ENREF_9)). Pseudobulk samples were created by pooling raw read counts across cells for each syngraft or allograft replicate sample (stored in the Seurat object’s metadata). Differential expression analysis of pseudobulk samples was further performed using DESeq2([10](#_ENREF_10)) and the batches and conditions were also considered. PCA with hierarchical clustering was performed to determine the biological reproducibility of the sample replicates. *P* or adjusted *p* values and normalized read counts (including log2(fold changes)) were obtained from DESeq2 output. Genes with adjusted *p* < 0.05 and fold changes > 1.5 (allograft vs syngraft) were considered significant. This procedure was used to identify differential genes for the MMs, NEs, DCs, TCs, BCs, and NK cells, respectively. All results (including significant and insignificant genes) of the pseudobulk analysis are available in the supplemental **File S2**.

**Gene ontology and pathway enrichment**

The DAVID Functional Annotation Tool([11](#_ENREF_11)) was used for GO analyses of significant genes (using biological process terms) among different samples. To annotate the differential expression at a gene set level, the significant genes were studied using the gene set enrichment analysis (GSEA). The GSEA-related KEGG pathways were identified using the *gseKEGG* function of the clusterProfiler R package and then visualized using the *gseaplot2* function. In addition, the gene ontology (GO) terms and KEGG pathways associated with the marker genes were analyzed using the *compareCluster* function in the clusterProfiler R package. The results were further visualized in dot plots.

**Transcription factor analysis**

Single-cell regulatory network inference and clustering (SCENIC) that has been implemented in Python (pySCENIC)([12](#_ENREF_12)) was utilized to uncover the gene regulatory network (GRN) and identify which transcription factors (TFs) were activated in major cell types. Only cell types (MMs, DCs, and NEs) with significant expression changes between syngrafts and allografts were analyzed using the pySCENIC protocol([12](#_ENREF_12)). Briefly, the Seurat subsets were extracted as a count matrix in R, input in Python using *scanpy*, and then converted into a loom file using *numpy* and *loompy*. Subsequently, coexpression modules were inferred using a regression per-target approach (GRNBoost2). Next, the indirect targets were pruned from these modules using cis-regulatory motif discovery (cisTarget). The reference databases, including motif, TSS+/10 kb, TSS+/5 kb, and mouse TFs are available from <https://www.resources.aertslab.org/cistarget/>. Then, the activity of these regulons was quantified via an enrichment score for the regulon’s target genes (AUCell). The results were exported as a loom file and visualized using the SCENIC R package([13](#_ENREF_13)). Furthermore, the regulon specificity score (RSS)([14](#_ENREF_14)) for each TF was calculated to identify graft-specific regulons. To identify GRN-based cell states, the regulon activity score was binarized into "on"/"off" by using the *binarizeAUC* function. The resulting binarized activity matrix was used for performing a new 2D projection or clustering using the ComplexHeatmap R package. Finally, an incidence matrix of the AUCell score (TFs by potential target genes of interest) was created and visualized using the pheatmap R package. We only considered regulons with 10 genes or more.

**Trajectory pseudotime analysis**

Although we focused on the 3 days early after transplant rather than time series analysis, the cell differentiation states or trajectories also can be reconstructed by using a pseudotime algorithm such as Monocle([15](#_ENREF_15)). The cells that have plasticity during immune response such as (monocyte-macrophage differentiation and T cell activation) were extracted from the integrated Seurat object for further analysis using the Monocle v2 R package. Firstly, the RNA count and metadata were uploaded for the generation of a CellDataSet Object by using the *newCellDataSet* function. After estimating size factors and dispersions, we filtered out low-quality cells by setting the expression threshold as 0.1 and the minimal number of expressed cells as 10. Then, groups of cells were compared in myriad ways to identify differentially expressed genes, controlling for batch effects, cell types, or graft types. The single-cell trajectory was created by choosing genes that define a cell's progress, reducing the dimensionality of the data, and ordering the cells in pseudotime. The trajectory results were visualized using the *plot_cell_trajectory* function and colored according to the cell types or graft types of interest. Furthermore, branches appearing in trajectories were analyzed to understand how cells make fate choices through gene regulation. A Monocle test of branched expression analysis modeling (BEAM) was used to generate a table of significance scores for each gene that would be branch-dependent. The BEAM results were visualized using the *plot_genes_branched_heatmap* function. The cell state-dependent gene sets were analyzed using the DAVID Functional Annotation Tool to reveal the related GO biological process terms. The branch-specific genes of interest were visualized using the *plot_genes_branched_pseudotime* function.

**V(D)J clonotype analysis**

Full-length TCR/BCR V(D)J segments were enriched from amplified cDNA from the 5’v1 libraries via PCR amplification using the 10× Chromium Single-Cell V(D)J Enrichment kit according to the manufacturer’s instructions. Raw sequencing files were demultiplexed using *cellranger mkfastq* and FASTQ files were generated. TCR/BCR clonotype assignments were analyzed using Cell Ranger (v6.0) vdj pipeline with mm10 as a reference genome. Subsequently, the cell ranger output including TCR/BCR clonotype frequency and barcode information was obtained. For comparing the difference between allografts and syngrafts, the TCR or BCR outputs for each dataset were aggregated using the *cellranger aggr* function. Finally, a Vloupe file was generated and visualized in Loupe V(D)J Browser v4.0.0 offered by 10× Genomics. The number and frequency information of V(D)J gene recombination at TCR (TRA-TRB pair) or BCR (IGH-IGK/IGL pair) was presented in the plots. If one clonotype was present in at least two cells, cells harboring this clonotype were considered to be clonally expanded and the number of cells with such pairs indicated the degree of clonality of the clonotype. All results of the clonotype analysis are available in the supplemental **File S3**.

**Cell-cell communication analysis**

The CellChat([16](#_ENREF_16)) toolkit was used to identify major signaling changes as well as conserved and cell-specific signaling within intercellular communication. The integrated Seurat object was split into two subsets-syngraft and allograft to uncover immune rejection-dependent pathways. The two datasets were converted into a CellChat object using the *createCellChat* function respectively. And then the two CellChat objects were merged using the *mergeCellChat* function for further analysis. Following the CellChat pipeline, the profiles of intercellular communications such as the incoming and outgoing signaling in cells and ligand-receptor pairs were visualized using circle plot, chord diagram, scatter plot, bubble plot, or heatmap.

**Western blotting**

Western blotting was performed according to our previous publications([17](#_ENREF_17), [18](#_ENREF_18)). Briefly, the heart tissues were harvested from various mice with heart transplantation and then homogenized with the SDS cell/tissue lysis buffer (Cell Signaling Technology). After centrifugation at 12,000rpm for 15min, the supernatant was taken and aliquoted in new tubes. The lysate was used immediately for detecting the protein concentration using the Bradford assay or stored at -80 °C until ready to use. After electrophoresis in precast protein gels (Thermo Fisher Scientific), transformation on PVDF membranes (Thermo Fisher Scientific), and immunoblotting with antibodies against Nlrp3 (Cell Signaling Technology #15101), Il1β (Cell Signaling Technology #31202), caspase-1 (Cell Signaling Technolog #24232), and β-actin (Cell Signaling Technology #4967) the samples on the PVDF membranes were visualized using an enhanced chemiluminescence system (Thermo Fisher Scientific), exposed to X-ray film, and then quantified by a laser scanner.

**Pharmacological intervention**

Fludarabine (Tocris) or VX765 (Tocris) was dissolved in a vehicle solvent (10% DMSO, 30% PEG300, and 50% ddH_2_O) respectively. Before allogeneic heart transplant for 2 hours, the recipients were administrated with Fludarabine (40mg/kg) or VX765 (50mg/kg) via intraperitoneal injections. The same volume of vehicle solvent was injected into other recipients with allografts as a control group. The recipients were continuously treated with the chemicals and once-daily administrated with the same amount of drug vehicle, Fludarabine, or VX765 after the surgery for one week.

**Heart function analysis**

The survival of transplanted hearts in the abdomen was determined by daily palpation and binocular inspection. The complete cessation of heartbeats indicated severe rejection as the endpoint in this study, and the survival time for the donor's hearts was recorded. In addition, after one week of surgery, the graft’s contractility function was assessed by a catheter approach as previously reported([19](#_ENREF_19)). Left ventricle systolic pressure (LVSP), left ventricle end-diastolic pressure (LVEDP), the maximal rate of change of the left ventricular pressure (±dp/dt), and the heart rate were recorded after 10 min of restoring the sinus rhythm, using a Millar pressure-volume (PV) catheter, and were analyzed using LabChart reader, as previously described([19](#_ENREF_19), [20](#_ENREF_20)). Briefly, 6-0 sutures were used to make a pursing string suture on the donor heart’s apex, then a 22 G needle was used to make a hole in the middle of the purse. The tip of the Mikro-Tip pressure catheter was placed into the left ventricle via the hole, and the purse-string suture was tightened. LabChart software was used to record and analyze the data once heart-beating was stable.

**Histological analysis**

The graft infiltration and rejection were confirmed by histological assay. The graft samples from recipients were collected after surgery for one week and perfused with 1% heparin in PBS to pump out the blood from the vessel lumen or ventricular chambers. Subsequently, the graft samples were fixed in a 10% neutral buffered formalin solution (Sigma). Tissue blocks were embedded in paraffin for sectioning. After deparaffinization and rehydration, the sections were stained with hematoxylin-eosin (Beyotime) according to the manufacturer’s protocol.

**Supplemental tables:**

**Table S1. Summary of Cell Ranger count results**

|  | Syn-B1 | Syn-B2 | Allo-B1 | Allo-B2 |
| --- | --- | --- | --- | --- |
| Estimated Number of Cells | 6,610 | 8,738 | 7,399 | 7,345 |
| Mean Reads per Cell | 89,926 | 56,611 | 82,346 | 63,403 |
| Median Genes per Cell | 2,362 | 2,578 | 2,093 | 2,644 |
| Valid Barcodes | 89.1% | 96.2% | 88.1% | 96.1% |
| Valid UMIs | 99.8% | 99.9% | 99.8% | 99.9% |
| Sequencing Saturation | 80.1% | 59.9% | 81.9% | 61.8% |
| Median UMI Counts per Cell | 9,397 | 10,049 | 7,365 | 10,833 |
| Reads Mapped Confidently to Genome | 85.5% | 82.7% | 83.6% | 83.4% |
| Reads Mapped Confidently to Transcriptome | 70.0% | 61.3% | 67.1% | 62.9% |

**Table S2. Cell Ranger pipeline showing the batch effect of the heart grafts**

|  | Syn-B1+B2 | Allo-B1+B2 |
| --- | --- | --- |
| Estimated Number of Cells | 15,348 | 14,744 |
| Mean Reads per Cell | 70,959 | 72,910 |
| Median UMI Counts per Cell | 9,760 | 9,008 |
| Fraction of Reads Kept | Syn-B1: 100.0%;  Syn-B2: 100.0% | Allo-B1: 100.0%;  Allo-B2: 100.0% |
| Batch Effect Score before Correction | 1.81 | 1.79 |
| Batch Effect Score after Correction | 1.44 | 1.39 |

**Table S3. Cell number and proportion of each cluster with cell annotation.**

| Cluster | ImmuGen  (cell subtypes) | Syn-B1 | | Syn-B2 | | Allo-B1 | | Allo-B2 | |
| --- | --- | --- | --- | --- | --- | --- | --- | --- | --- |
|  |  | # | % | # | % | # | % | # | % |
| 17 | B cells (B.FrF) | 27 | 0.45 | 38 | 0.49 | 69 | 1.03 | 39 | 0.59 |
| 18 | DC (DC.103+11B-) | 20 | 0.33 | 46 | 0.59 | 21 | 0.31 | 63 | 0.96 |
| 6 | DC (DC.103-11B+24+) | 162 | 2.67 | 262 | 3.38 | 240 | 3.59 | 246 | 3.75 |
| 16 | Endothelial cells (BEC) | 93 | 1.54 | 56 | 0.72 | 68 | 1.02 | 39 | 0.59 |
| 10 | Fibroblasts (FI) | 234 | 3.86 | 28 | 0.36 | 175 | 2.62 | 33 | 0.50 |
| 13 | Macrophages (MF.103-11B+24-) | 63 | 1.04 | 151 | 1.95 | 86 | 1.29 | 73 | 1.11 |
| 0 | Macrophages (MFIO5.II+480INT) | 1103 | 18.21 | 1562 | 20.15 | 1458 | 21.84 | 1211 | 18.44 |
| 2 | Macrophages (MFIO5.II+480INT) | 909 | 15.00 | 1203 | 15.52 | 749 | 11.22 | 938 | 14.29 |
| 7 | Macrophages (MFIO5.II+480INT) | 262 | 4.32 | 178 | 2.30 | 145 | 2.17 | 146 | 2.22 |
| 11 | Macrophages (MFIO5.II+480INT) | 115 | 1.90 | 107 | 1.38 | 131 | 1.96 | 45 | 0.69 |
| 12 | Macrophages (MFIO5.II-480INT) | 146 | 2.41 | 111 | 1.43 | 63 | 0.94 | 68 | 1.04 |
| 9 | Monocytes (MO.6C+II-) | 84 | 1.39 | 177 | 2.28 | 93 | 1.39 | 117 | 1.78 |
| 4 | Monocytes (MO.6C+II-) | 516 | 8.52 | 1004 | 12.95 | 840 | 12.58 | 871 | 13.27 |
| 5 | Monocytes (MO.6C+II-) | 150 | 2.48 | 285 | 3.68 | 592 | 8.87 | 558 | 8.50 |
| 14 | Monocytes (MO.6C-IIINT) | 46 | 0.76 | 115 | 1.48 | 62 | 0.93 | 93 | 1.42 |
| 1 | Neutrophils (GN.ARTH) | 1039 | 17.15 | 1259 | 16.24 | 883 | 13.23 | 1057 | 16.10 |
| 3 | Neutrophils (GN.Thio) | 999 | 16.49 | 971 | 12.52 | 747 | 11.19 | 713 | 10.86 |
| 15 | NK cells (NK.DAP10-) | 31 | 0.51 | 52 | 0.67 | 84 | 1.26 | 93 | 1.42 |
| 19 | T cells (T.8EFF.OT1LISO) | 1 | 0.02 | 9 | 0.12 | 23 | 0.34 | 10 | 0.15 |
| 8 | T cells (T.8EFF.TBET-.OT1LISOVA) | 58 | 0.96 | 139 | 1.79 | 147 | 2.20 | 153 | 2.33 |

**Table S4. Representative marker genes of identified cell subtypes.**

| Cell subtype | Marker gene | Manual annotation |
| --- | --- | --- |
| MM1 | *Gpnmb*, *Fabp5*, *Selenop* | Lipid transport, anti-inflammation([21](#_ENREF_21), [22](#_ENREF_22)) |
| MM2 | *Ccl2*, *Hmox1*, *Mrc1* (Cd206) | Anti-inflammation |
| MM3 | *Ly6c2*, *Ccr2*, *Chil3* | Markers of classical monocytes |
| MM4 | *Cd74*, *H2-Aa*, *H2-Ab1* | Antigen processing/presentation |
| MM5 | *Stmn1*, *Pclaf*, *Top2a* | Cell cycling/proliferation |
| MM6 | *Irf7*, *Isg15*, *Ifi209* | Pro-inflammatory IFN signaling |
| MM7 | *Fn1*, *Ccl9*, *Arg1* | Anti-inflammation |
| MM8 | *Ube2c*, *Birc5*, *Cenpa* | Cell cycling/proliferation |
| MM9 | *Lars2*, *Gm26917*, *Gm42418*, *Neat1* | Inflammasome activation([23](#_ENREF_23)) |
| MM10 | *Adgre4*, *Treml4*, *Ear2* | Pro-inflammation |
| DC1 | *Klrd1,* *Cd7* | For NK cell activation([24](#_ENREF_24)) |
| DC2 | *Batf3*, *Cd103*, *Xcr1* | Markers of type 1 conventional dendritic cells |
| NE1 | *Retnlg*, *Lcn2*, *Asprv1* | Neutrophil maturation, chronic inflammation([25](#_ENREF_25), [26](#_ENREF_26)) |
| NE2 | *Tnf*, *Il1b*, *Nlrp3* | Acute inflammation, inflammasome formation([27](#_ENREF_27)) |
| NK | *Gzma*, *Gzmb*, *Prf1*, *Klre1,* *Klrb1c* | Cytolysis, cytotoxicity |
| BC | *Cd79a*, *Igkc*, *Ebf1* | Early B-lineage formation |
| TC1 | *Cd3d, C3e, Il7r* | Markers of naive T cells |
| TC2 | *Cd3d, Cd8a*, *Stmn1*, *Mki67*, *Pclaf* | Cytotoxicity, cell cycling, proliferation |
| EC | *Fabp4, Cav1,Cdh5, Flt1, Pecam1* | Sprouting angiogenesis |
| FB | *Col3a1, Col1a1, Dcn, Mfap5, Postn* | Collagen fibril organization |

**Supplemental references:**

1. M. Okabe, M. Ikawa, K. Kominami, T. Nakanishi and Y. Nishimune: 'Green mice' as a source of ubiquitous green cells. *FEBS Lett*, 407(3), 313-9 (1997) doi:10.1016/s0014-5793(97)00313-x

2. Y. Hao, S. Hao, E. Andersen-Nissen, W. M. Mauck, 3rd, S. Zheng, A. Butler, M. J. Lee, A. J. Wilk, C. Darby, M. Zager, P. Hoffman, M. Stoeckius, E. Papalexi, E. P. Mimitou, J. Jain, A. Srivastava, T. Stuart, L. M. Fleming, B. Yeung, A. J. Rogers, J. M. McElrath, C. A. Blish, R. Gottardo, P. Smibert and R. Satija: Integrated analysis of multimodal single-cell data. *Cell* (2021) doi:10.1016/j.cell.2021.04.048

3. C. S. McGinnis, L. M. Murrow and Z. J. Gartner: DoubletFinder: Doublet Detection in Single-Cell RNA Sequencing Data Using Artificial Nearest Neighbors. *Cell Syst*, 8(4), 329-337 e4 (2019) doi:10.1016/j.cels.2019.03.003

4. T. Stuart, A. Butler, P. Hoffman, C. Hafemeister, E. Papalexi, W. M. Mauck, 3rd, Y. Hao, M. Stoeckius, P. Smibert and R. Satija: Comprehensive Integration of Single-Cell Data. *Cell*, 177(7), 1888-1902 e21 (2019) doi:10.1016/j.cell.2019.05.031

5. L. Zappia and A. Oshlack: Clustering trees: a visualization for evaluating clusterings at multiple resolutions. *Gigascience*, 7(7) (2018) doi:10.1093/gigascience/giy083

6. D. Aran, A. P. Looney, L. Liu, E. Wu, V. Fong, A. Hsu, S. Chak, R. P. Naikawadi, P. J. Wolters, A. R. Abate, A. J. Butte and M. Bhattacharya: Reference-based analysis of lung single-cell sequencing reveals a transitional profibrotic macrophage. *Nat Immunol*, 20(2), 163-172 (2019) doi:10.1038/s41590-018-0276-y

7. T. S. Heng and M. W. Painter: The Immunological Genome Project: networks of gene expression in immune cells. *Nat Immunol*, 9(10), 1091-4 (2008) doi:10.1038/ni1008-1091

8. V. Y. Kiselev, A. Yiu and M. Hemberg: scmap: projection of single-cell RNA-seq data across data sets. *Nat Methods*, 15(5), 359-362 (2018) doi:10.1038/nmeth.4644

9. R. A. Amezquita, A. T. L. Lun, E. Becht, V. J. Carey, L. N. Carpp, L. Geistlinger, F. Marini, K. Rue-Albrecht, D. Risso, C. Soneson, L. Waldron, H. Pages, M. L. Smith, W. Huber, M. Morgan, R. Gottardo and S. C. Hicks: Orchestrating single-cell analysis with Bioconductor. *Nat Methods*, 17(2), 137-145 (2020) doi:10.1038/s41592-019-0654-x

10. M. I. Love, W. Huber and S. Anders: Moderated estimation of fold change and dispersion for RNA-seq data with DESeq2. *Genome Biol*, 15(12), 550 (2014) doi:10.1186/s13059-014-0550-8

11. W. Huang da, B. T. Sherman and R. A. Lempicki: Systematic and integrative analysis of large gene lists using DAVID bioinformatics resources. *Nat Protoc*, 4(1), 44-57 (2009) doi:10.1038/nprot.2008.211

12. B. Van de Sande, C. Flerin, K. Davie, M. De Waegeneer, G. Hulselmans, S. Aibar, R. Seurinck, W. Saelens, R. Cannoodt, Q. Rouchon, T. Verbeiren, D. De Maeyer, J. Reumers, Y. Saeys and S. Aerts: A scalable SCENIC workflow for single-cell gene regulatory network analysis. *Nat Protoc*, 15(7), 2247-2276 (2020) doi:10.1038/s41596-020-0336-2

13. S. Aibar, C. B. Gonzalez-Blas, T. Moerman, V. A. Huynh-Thu, H. Imrichova, G. Hulselmans, F. Rambow, J. C. Marine, P. Geurts, J. Aerts, J. van den Oord, Z. K. Atak, J. Wouters and S. Aerts: SCENIC: single-cell regulatory network inference and clustering. *Nat Methods*, 14(11), 1083-1086 (2017) doi:10.1038/nmeth.4463

14. S. Suo, Q. Zhu, A. Saadatpour, L. Fei, G. Guo and G. C. Yuan: Revealing the Critical Regulators of Cell Identity in the Mouse Cell Atlas. *Cell Rep*, 25(6), 1436-1445 e3 (2018) doi:10.1016/j.celrep.2018.10.045

15. C. Trapnell, D. Cacchiarelli, J. Grimsby, P. Pokharel, S. Li, M. Morse, N. J. Lennon, K. J. Livak, T. S. Mikkelsen and J. L. Rinn: The dynamics and regulators of cell fate decisions are revealed by pseudotemporal ordering of single cells. *Nat Biotechnol*, 32(4), 381-386 (2014) doi:10.1038/nbt.2859

16. S. Jin, C. F. Guerrero-Juarez, L. Zhang, I. Chang, R. Ramos, C. H. Kuan, P. Myung, M. V. Plikus and Q. Nie: Inference and analysis of cell-cell communication using CellChat. *Nat Commun*, 12(1), 1088 (2021) doi:10.1038/s41467-021-21246-9

17. J. L. Liang, D. Z. Xiao, X. Y. Liu, Q. X. Lin, Z. X. Shan, J. N. Zhu, S. G. Lin and X. Y. Yu: High glucose induces apoptosis in AC16 human cardiomyocytes via macrophage migration inhibitory factor and c-Jun N-terminal kinase. *Clin Exp Pharmacol Physiol*, 37(10), 969-73 (2010) doi:10.1111/j.1440-1681.2010.05420.x

18. J. L. Liang, Z. K. Feng, X. Y. Liu, Q. X. Lin, Y. H. Fu, Z. X. Shan, J. N. Zhu, S. G. Lin and X. Y. Yu: Effect of impaired glucose tolerance on cardiac dysfunction in a rat model of prediabetes. *Chin Med J (Engl)*, 124(5), 734-9 (2011) doi:10.1111/j.1440-1681.2010.05420.x %/ (c) 2010 The Authors. Clinical and Experimental Pharmacology and Physiology (c) 2010 Blackwell Publishing Asia Pty Ltd.

19. P. Pacher, T. Nagayama, P. Mukhopadhyay, S. Bátkai and D. A. Kass: Measurement of cardiac function using pressure-volume conductance catheter technique in mice and rats. *Nat Protoc*, 3(9), 1422-34 (2008) doi:10.1038/nprot.2008.138

20. Z. Wu, J. Liang, W. Huang, L. Jiang, C. Paul, B. Lin, J. Zheng and Y. Wang: Prompt Graft Cooling Enhances Cardioprotection during Heart Transplantation Procedures through the Regulation of Mitophagy. *Cells*, 10(11) (2021) doi:10.3390/cells10112912

21. V. M. Ripoll, K. M. Irvine, T. Ravasi, M. J. Sweet and D. A. Hume: Gpnmb is induced in macrophages by IFN-gamma and lipopolysaccharide and acts as a feedback regulator of proinflammatory responses. *J Immunol*, 178(10), 6557-66 (2007) doi:10.4049/jimmunol.178.10.6557

22. Y. Guo, Y. Liu, S. Zhao, W. Xu, Y. Li, P. Zhao, D. Wang, H. Cheng, Y. Ke and X. Zhang: Oxidative stress-induced FABP5 S-glutathionylation protects against acute lung injury by suppressing inflammation in macrophages. *Nat Commun*, 12(1), 7094 (2021) doi:10.1038/s41467-021-27428-9

23. P. Zhang, L. Cao, R. Zhou, X. Yang and M. Wu: The lncRNA Neat1 promotes activation of inflammasomes in macrophages. *Nat Commun*, 10(1), 1495 (2019) doi:10.1038/s41467-019-09482-6

24. A. Crinier, P. Milpied, B. Escaliere, C. Piperoglou, J. Galluso, A. Balsamo, L. Spinelli, I. Cervera-Marzal, M. Ebbo, M. Girard-Madoux, S. Jaeger, E. Bollon, S. Hamed, J. Hardwigsen, S. Ugolini, F. Vely, E. Narni-Mancinelli and E. Vivier: High-Dimensional Single-Cell Analysis Identifies Organ-Specific Signatures and Conserved NK Cell Subsets in Humans and Mice. *Immunity*, 49(5), 971-986 e5 (2018) doi:10.1016/j.immuni.2018.09.009

25. R. F. Whittaker Hawkins, A. Patenaude, A. Dumas, R. Jain, Y. Tesfagiorgis, S. Kerfoot, T. Matsui, M. Gunzer, P. E. Poubelle, C. Larochelle, M. Pelletier and L. Vallières: ICAM1+ neutrophils promote chronic inflammation via ASPRV1 in B cell-dependent autoimmune encephalomyelitis. *JCI insight*, 2(23), e96882 (2017) doi:10.1172/jci.insight.96882

26. D. M. Calcagno, C. Zhang, A. Toomu, K. Huang, V. K. Ninh, S. Miyamoto, A. D. Aguirre, Z. Fu, J. Heller Brown and K. R. King: SiglecF(HI) Marks Late-Stage Neutrophils of the Infarcted Heart: A Single-Cell Transcriptomic Analysis of Neutrophil Diversification. *J Am Heart Assoc*, 10(4), e019019 (2021) doi:10.1161/jaha.120.019019

27. M. D. McGeough, A. Wree, M. E. Inzaugarat, A. Haimovich, C. D. Johnson, C. A. Pena, R. Goldbach-Mansky, L. Broderick, A. E. Feldstein and H. M. Hoffman: TNF regulates transcription of NLRP3 inflammasome components and inflammatory molecules in cryopyrinopathies. *J Clin Invest*, 127(12), 4488-4497 (2017) doi:10.1172/JCI90699
